# Evaluation of robenidine analog NCL195 as a novel broad-spectrum antibacterial agent

**DOI:** 10.1371/journal.pone.0183457

**Published:** 2017-09-05

**Authors:** Abiodun D. Ogunniyi, Manouchehr Khazandi, Andrew J. Stevens, Sarah K. Sims, Stephen W. Page, Sanjay Garg, Henrietta Venter, Andrew Powell, Karen White, Kiro R. Petrovski, Geraldine Laven-Law, Eliane G. Tótoli, Hérida R. Salgado, Hongfei Pi, Geoffrey W. Coombs, Dean L. Shinabarger, John D. Turnidge, James C. Paton, Adam McCluskey, Darren J. Trott

**Affiliations:** 1 Australian Centre for Antimicrobial Resistance Ecology, School of Animal and Veterinary Sciences, The University of Adelaide, Roseworthy, South Australia, Australia; 2 Chemistry, School of Environmental & Life Sciences, The University of Newcastle, Callaghan, New South Wales, Australia; 3 School of Animal and Veterinary Sciences, The University of Adelaide, Roseworthy, South Australia, Australia; 4 Neoculi Pty Ltd, Burwood, Victoria, Australia; 5 Centre for Pharmaceutical Innovation and Development, School of Pharmacy and Medical Sciences, Sansom Institute for Health Research, University of South Australia, Adelaide, South Australia, Australia; 6 School of Pharmacy and Medical Sciences, Sansom Institute for Health Research, University of South Australia, Adelaide, South Australia, Australia; 7 Centre for Drug Candidate Optimisation, Monash Institute of Pharmaceutical Sciences, Monash University, Parkville, Victoria, Australia; 8 Department of Drugs and Medicines, School of Pharmaceutical Sciences, São Paulo State University, Araraquara, São Paulo, Brazil; 9 Department of Microbiology, Path West Laboratory Medicine-WA, Fiona Stanley Hospital, Murdoch, Western Australia, Australia; 10 School of Veterinary and Life Sciences, Murdoch University, Murdoch, Western Australia, Australia; 11 Micromyx LLC, Kalamazoo, Michighan, United States of America; 12 Department of Molecular and Cellular Biology, School of Biological Sciences, The University of Adelaide, Adelaide, South Australia, Australia; 13 Research Centre for Infectious Diseases, Department of Molecular and Cellular Biology, School of Biological Sciences, The University of Adelaide, Adelaide, South Australia, Australia; Universite Paris-Sud, FRANCE

## Abstract

The spread of multidrug resistance among bacterial pathogens poses a serious threat to public health worldwide. Recent approaches towards combating antimicrobial resistance include repurposing old compounds with known safety and development pathways as new antibacterial classes with novel mechanisms of action. Here we show that an analog of the anticoccidial drug robenidine (4,6-bis(2-((*E*)-4-methylbenzylidene)hydrazinyl)pyrimidin-2-amine; NCL195) displays potent bactericidal activity against *Streptococcus pneumoniae* and *Staphylococcus aureus* by disrupting the cell membrane potential. NCL195 was less cytotoxic to mammalian cell lines than the parent compound, showed low metabolic degradation rates by human and mouse liver microsomes, and exhibited high plasma concentration and low plasma clearance rates in mice. NCL195 was bactericidal against *Acinetobacter* spp and *Neisseria meningitidis* and also demonstrated potent activity against *A*. *baumannii*, *Pseudomonas aeruginosa*, *Escherichia coli*, *Klebsiella pneumoniae* and *Enterobacter* spp. in the presence of sub-inhibitory concentrations of ethylenediaminetetraacetic acid (EDTA) and polymyxin B. These findings demonstrate that NCL195 represents a new chemical lead for further medicinal chemistry and pharmaceutical development to enhance potency, solubility and selectivity against serious bacterial pathogens.

## Introduction

Infectious diseases particularly lower respiratory infections caused by bacterial and viral pathogens are the third leading cause of death [1] and the second leading cause of disability-adjusted life years, worldwide [2]. *Streptococcus pneumoniae* (the pneumococcus) and *Staphylococcus aureus* (‘golden staph’) are two such bacterial pathogens. The pneumococcus accounts for an estimated one million deaths annually in developing countries, and causes a broad spectrum of diseases including pneumonia, meningitis, bacteremia and otitis media (OM), particularly in children under five years of age, the elderly and immunocompromised individuals [3]. Although the current capsular-based pneumococcal vaccines have been effective in preventing pneumococcal sepsis and meningitis in developed countries, the vaccines induce strictly serotype-specific protection against a limited subset of pneumococcal types and furthermore are generally not affordable in developing countries. In addition, there is evident replacement of carriage and disease burden by non-vaccine serotypes following immunization [4, 5]. In developed countries, where effective antimicrobial therapy is readily accessible, deaths from pneumococcal disease occur primarily among people over 60 years of age, with case fatality rates of 10–20% for pneumonia and up to 40% for bacteremia [3]. In contrast, *S*. *aureus* is the second most clinically important antibiotic-resistant bacterial pathogen in developed countries behind *E*. *coli*, and a major public health concern due to the increasing prevalence of methicillin-resistant *S*. *aureus* (MRSA) in hospitals, among hospital workers and within the community [6–9]. Median direct medical costs associated with MRSA sepsis have been estimated at $12,078 US per patient and are approximately1.3 times higher than costs associated with susceptible *S*. *aureus* infection [10].

Effective treatment of Gram-positive bacterial infections remains hampered by the alarming increase in prevalence of antibiotic-resistant bacteria, as well as accelerated global spread of bacterial clones resistant to multiple antimicrobial agents [11, 12]. For pneumococcal disease, the problem is compounded by the highly transformable nature of this organism, enhancing genetic exchanges through horizontal gene transfer [13]. Moreover, pneumococcal OM accounts for more antibiotic prescriptions than any other infection [14, 15], driving selection for resistance amongst diverse bacteria, significantly affecting health-care costs in developed countries [$2.88 billion p.a. for USA] [16]. Recently, novel antimicrobials such as third-generation fluoroquinolones (e.g. moxifloxacin) and cyclic lipopeptides (e.g. daptomycin) have been shown to be effective against *S*. *pneumoniae in vitro* and in mouse models of infection [17–19]. However, there are reports of increasing frequency of *S*. *pneumoniae* resistance to the fluoroquinolones [20]. For MRSA, resistance or reduced susceptibility to multiple agents including vancomycin and daptomycin, is frequently reported in hospital-associated MRSA (HA-MRSA) [21]. Although community-associated MRSA (CA-MRSA) are generally susceptible to most drug classes over time they have acquired additional antimicrobial resistance genes. Furthermore, CA-MRSA are generally more virulent with higher mortality rates compared to HA-MRSA [22, 23]. Certain MRSA clones have recently become host-adapted in animals, furthering the spread of the staphylococcal cassette chromosome *mec* (SCC*mec*) within the environment [24]. In some countries, up to 54% of *K*. *pneumoniae* cases have been reported as carbapenem-resistant, the current last resort treatment for severe Gram-negative infections [25, 26]. Adding to this dire situation, the transferable colistin resistance determinant (MCR-1) is now widely distributed amongst both animal and human Gram-negative pathogens and commensals, resulting in some Gram-negative pathogens developing pan-resistance to all registered compounds [27, 28]. Therefore, the discovery and development of new broad-spectrum antimicrobials with activity against pan-resistant *Enterobacteriaceae* causing sepsis is one of the highest global priorities [29–31].

In 2010, the Infectious Diseases Society of America has called for 10 new antimicrobial agents to be registered by 2020 [32]. Since that call, all new registered agents except one (fidaxomicin) have been developed from existing drug classes, against Gram-positive organisms (telavancin, dalbavancin, tedizolid, oritavancin) or Gram-negative organisms (ceftolozane-tazobactam, ceftazidime-avibactam) [33–36]. Robenidine (2,2'-bis[(4-chlorophenyl)methylene] carbonimidic dihydrazide hydrochloride) is an anticoccidial agent which has been used worldwide since the early 1970s to prevent coccidian infections of poultry and rabbits [37]. However, there have been no in-depth *in vivo* evaluations of this compound or its chemical class as potential antibacterial agents. This presents an attractive prospect for testing the antibacterial activity of robenidine, an agent with known safety and long history of oral use in avian species and an oral LD_50_ of 150 mg/kg in mice [38], by expanding its chemical space with further medicinal chemistry for potential development as a parenteral or topically administered drug [39–41].

## Materials and methods

### Antimicrobial agents and medicinal chemistry

Analytical grade aminoguanidine robenidine (NCL812) [42] and two of its analogs (NCL195 and NCL219) were synthesized in house at The University of Newcastle and stored in a sealed sample container out of direct light at 4°C at the study site at the Infectious Diseases Laboratory, Roseworthy campus, The University of Adelaide. Ampicillin (β-lactam broad-spectrum antibiotic) was purchased from Sigma-Aldrich (Australia), while daptomycin (in pharmaceutically active form) was purchased from Cubist. Stock solutions (containing 25.6 mg/ml of each compound in DMSO) were prepared and stored in 1 ml aliquots at -80°C and defrosted immediately prior to use. Ethylenediaminetetraacetic acid (EDTA, Disodium salt, CAS Number: 6381-92-6) was purchased from Chem-Supply Pty Ltd, South Australia and was dissolved in water to 200 mM. Polymyxin B sulfate (CAS Number: 1405-20-5) was purchased from Sigma-Aldrich.

#### Synthesis of NCL195 (4,6-bis(2-((E)-4-methylbenzylidene)hydrazinyl)pyrimidin-2-amine)

A suspension of 2-amino-4,6-dihydrazinopyrimidine (58.9 mg, 0.380 mmol) and 4-methylbenzaldehyde (0.10 ml, 100 mg, 0.832 mmol, 2.19 eq.) in EtOH (4 ml) was heated at reflux for 16 h. The reaction mixture was cooled to ambient temperature before collecting the pellet-like precipitate, washing with diethyl ether (20 ml). The ‘pellets’ were then crushed and the solid further washed with diethyl ether (10 ml) to afford the pyrimidine (85.8 mg, 63%) as a white ‘fluffy’ powder. ^1^H NMR ((CD_3_)_2_SO, 400 MHz) δ 10.51 (s, 2H), 8.00 (s, 2H), 7.54 (d, *J* = 8.1 Hz, 4H), .26 (d, *J* = 8.0 Hz, 4H), 6.27 (s, 1H), 5.78 (s, 2H), 2.34 (s,6H); ^13^C NMR ((CD_3_)_2_SO, 101 MHz) δ 162.8 (2C), 162.6 (2C), 140.1 (2C), 138.4 (2C), 132.5 (2C), 129.4 (4C), 126.0 (4C), 73.3, 21.0 (6C).

#### *Synthesis of NCL219*. (*E*)-*N*'-((*E*)-1-(4-(*tert*-butyl)phenyl)ethylidene)-2-(1-(4-(*tert*-butyl)phenyl)ethylidene)hydrazine-1-carboximidhydrazide)

A suspension of *N*,*N*-diaminoguanidine hydrochloride (47.7 mg, 0.380 mmol) and 4′-(*tert*-butyl)acetophenone (134 mg, 0.760 mmol, 2 eq.) and in EtOH was heated at reflux for 16 h. The mixture was cooled and diluted with Et_2_O to effect crystallization. The resulting precipitate was collected and washed with Et_2_O 920 ml) to afford the carbonimidic dihydrazide (134 mg, 84%) as a yellow crystalline solid. ^1^H NMR ((CD_3_)_2_SO, 400 MHz) δ 11.74 (s, 1H), 8.60 (s, 1H), 7.95 (d, *J* = 8.6 Hz, 2H), 7.45 (d, *J* = 8.6 Hz, 2H), 2.42 (s, 3H), 1.31 (s, 9H). ^13^C NMR ((CD_3_)_2_SO, 101 MHz) δ 154.1, 153.3, 152.6, 134.0, 126.8, 125.0, 34.5, 30.9, 14.9.

### Bacterial strains and growth conditions

The *S*. *pneumoniae*, *S*. *aureus* and VRE isolates used in this study are listed in S1, S2 and S3 Tables in Supporting information. Isolates were cultured on Horse Blood Agar (HBA) plates and incubated at 37°C, for 18 h. The pneumococci isolates were incubated in 5% CO_2_. The NCL812, NCL195 and NCL219 minimum inhibitory concentration (MIC) was performed on all isolates.

Pneumococcal serotype-specific capsule production was confirmed on all pneumococcal isolates by the Quellung reaction [43]. The minimum bactericidal concentration (MBC), kill kinetics, TEM and efficacy testing experiments was performed on *S*. *pneumoniae* D39 (NCTC7466), a well-documented laboratory strain with well-defined and predictable *in vitro* characteristics and *in vivo* pathogenicity profiles [44, 45]. MBCs, kill kinetics and point of resistance assays were performed on *S*. *aureus* Xen29 (luminescent ATCC 12600 *S*. *aureus* strain).

### MIC determination

MICs for NCL812 and its analogs (serial two-fold dilutions commencing at 128 μg/ml) were determined (in duplicate) in round bottom 96-well microtitre trays (Sarstedt 82.1582.001), using the broth micro-dilution method recommended by the Clinical and Laboratory Standards Institute (CLSI) [46]. Luria Bertani (LB) broth (Oxoid, Australia) [supplemented with 3% lysed horse blood and 5% horse serum for *S pneumoniae* experiments] was used instead of cation-adjusted Mueller-Hinton broth (CAMHB) as it was shown previously that NCL812 can chelate calcium ions [47]. In addition, serial two-fold dilutions of the NCL compounds were performed in 100% DMSO, with 1 μl added to each well, as the compounds are hydrophobic [42]. The MIC for ampicillin or daptomycin against each *S*. *pneumoniae*, *S*. *aureus* or VRE isolate was determined for each test run as an internal quality control. MIC_50_, MIC_90_ and MIC range for NCL812, NCL195 and NCL219 were calculated against each *S*. *pneumoniae*, *S*. *aureus* or VRE isolate. To assess the potential activity of NCL195 against Gram-negative bacteria, MICs against *Acinetobacter* spp, *E*. *coli*, *K*. *pneumoniae*, *P*. *aeruginosa*, *Proteus* spp and *Neisseria meningitidis* were performed in the presence or absence of 0.08 mM-20 mM EDTA in a standard checkerboard assay [48].

### Kill kinetics

Kill kinetic assays on *S*. *pneumoniae* D39 were performed (in duplicate) essentially as described for MIC determination, with the exception that the starting concentration of each NCL compound was 64 μg/ml, and the assay was performed using 200 μl volumes in a round bottom 96-well microtitre tray (Sarstedt 82.1582.001). The tray was covered with Breathe-Easy sealing membrane (Z380059, Sigma-Aldrich) and incubated for 16 h overnight at 35°C in an EnSpire Multimode Plate Reader 2300 (PerkinElmer), with data collected every 20 min and 2 sec agitation at 60 rpm between data (*A*_600 nm_) acquisitions. The experiment was repeated using the luminescent D39LUX strain on a Cytation 5 Cell Imaging Multi-Mode Reader (BioTek) using IsoplateTM-96 F tray PerkinElmer, 6005020). Kill kinetic assays on *S*. *aureus* Xen29 was carried out essentially as described for MIC determination on Cytation 5 Cell Imaging Multi-Mode Reader.

### Minimum bactericidal concentration (MBC) determination

In order to determine the MBC of each compound against *S*. *pneumoniae* D39 or *S*. *aureus* Xen29, 10 **μ**l aliquots from each duplicate well from the MIC assays (starting from the MIC for each compound) was inoculated onto a HBA plate and incubated at 37°C (+ 5% CO_2_ for *S*. *pneumoniae* D39). Plates were examined at 24 and 48 h and the MBC was recorded as the lowest concentration of each test compound at which a 99.9% colony count reduction was observed on the plate [46].

### Time-dependent killing assays

Time kill assays were performed (in duplicate) essentially as described previously [42, 49] with slight modifications. Briefly, a few colonies of luminescent *S*. *pneumoniae* strain D39LUX or luminescent *S*. *aureus* ATCC 12600 (Xen29) from an overnight HBA were emulsified in normal saline and adjusted to *A*_600 nm_ = 0.10 (equivalent to approx. 5 × 10^7^ c.f.u. per ml) and the bacterial suspension further diluted 1:20 in saline. NCL195 was prepared in 10 ml volumes at 2× and 4× MIC (using 4× MIC of ampicillin and normal saline as controls) in either LB broth (for Xen29) or LB supplemented with 3% lysed horse blood and 5% horse serum (for D39). The compounds were serially diluted in 100% DMSO at 100× the final desired concentration and 100 μl of appropriate concentrations added to each 10 ml preparation. Duplicate cultures were incubated at 37°C (+ 5% CO_2_ for *S*. *pneumoniae* D39), with samples withdrawn at 0, 0.5, 1, 2, 4, 6, 8 and 24 h, serially diluted 10-fold and plated on HBA overnight at 37°C (+ 5% CO_2_ for *S*. *pneumoniae* D39) for bacterial enumeration.

### Point of resistance assay

To determine if *S*. *aureus* develops resistance to the NCL195, we performed 24 daily sequential culturing of *S*. *aureus* Xen29 in 2 ml LB broth the presence of 0.5×MIC, 0.75×MIC, 1×MIC, 1.5×MIC, 2×MIC, and 4×MIC of NCL195, using 1×MIC, 4×MIC and 8×MIC of daptomycin as control (in triplicates), essentially as described by Ling *et al*.[50]. After the 24^th^ passage, samples were centrifuged at 4,000 *× g* for 10 min and washed in 50 ml of phosphate buffered saline (PBS) twice to remove any residual antimicrobial, and/or bacterial end products and media. Washed bacteria were resuspended in PBS to *A*_600 nm_ = 0.1 and MICs were performed as described above.

### Effect of NCL812 on *S*. *pneumoniae* D39 and *S*. *aureus* ATCC29213 cell membrane

Morphological appearance and morphometric analysis of the cell membrane of *S*. *pneumoniae* D39 after exposure to either 1 μg/ml, 4 μg/ml or 16 μg/ml of NCL812 was determined using TEM, as described in **S1 File (Supplementary Methods)**. For *S*. *aureus* ATCC 29213, macromolecular (DNA, RNA, protein, cell wall, and lipid) synthesis inhibition studies were performed using concentrations of NCL812 that were equivalent to 0, 0.25, 0.5, 1, 2, 4 or 8-fold the MIC value (4 μg/ml), as detailed in **S1 File (Supplementary Methods).**

### Mechanism of action studies

To further examine the mechanism underlying the perturbation of the cell membrane of *S*. *pneumoniae* or *S*. *aureus* by the NCL compounds, the membrane potential of the cells was measured by fluorescence spectrometry using (DiOC_2_(3), essentially as described previously[51, 52]. Briefly, D39 cells from an overnight HBA plate were grown in Todd-Hewitt broth supplemented with 1% yeast extract (THY) at 37°C, 5% CO_2_ until *A*_600 nm_ = 0.5. The cells were centrifuged at 4,000 *× g* for 10 min at room temperature, washed twice in one culture volume of 50 mM potassium phosphate buffer (pH 7.0) and resuspended in the same buffer to *A*_600 nm_ = 10. For the fluorescence assay, 0.2 ml of this suspension was added to 1.8 ml of the same buffer in a quartz cuvette, the mixture was stirred gently for 5 min (with or without addition of 16 μg/ml of test compounds, using 16 μg/ml ampicillin as control). This concentration corresponds to 2×MIC of NCL812 and NCL195, and 4×MIC of NCL219 for *S*. *pneumoniae*. The cuvette was then placed in a LS 55 Fluorescence Spectrometer (PerkinElmer) set at Ex. 488 nm/Em. 620 nm, with excitation and emission slit widths at 5 nm and 7 nm, respectively). The background fluorescence of each suspension was followed for 1 min after which DiOC_2_(3) was added to a final concentration of 10 μM and the fluorescence monitored until it plateaued. Cells were then re-energized with 0.5% glucose and fluorescence further monitored until it plateaued, after which 10 μM of the proton ionophore carbonyl cyanide *m*-chlorophenyl hydrazone (CCCP) was added and fluorescence followed again until plateaued. A similar procedure was followed using *S*. *aureus* cells resuspended in 50 mM potassium phosphate buffer (pH 7.0) to *A*_600 nm_ = 5, using 16 μg/ml of test compounds, corresponding to 4×MIC of NCL812, 8×MIC NCL195, and 1×MIC of NCL219 for *S*. *aureus*. All assays were performed at least twice.

### Microsomal stability of NCL812, NCL195 and NCL219

The metabolic stability assay was performed by incubating 0.4 μg/ml of each test compound with human and mouse liver microsomes (Xenotech, Lot# 1210057 and 1310211, respectively) suspended in 0.1 M pH 7.4 phosphate buffer at 37°C and 0.4 mg/ml protein concentration. The metabolic reaction was initiated by the addition of an NADPH-regenerating system (i.e. NADPH is the cofactor required for CYP450-mediated metabolism) and quenched at various time points over a 60 min incubation period by the addition of acetonitrile containing diazepam as internal standard. Control samples (containing no NADPH) were included (and quenched at 2, 30 and 60 min) to monitor for potential degradation in the absence of cofactor. Test compound concentration *vs* time data were fitted to an exponential decay function to determine the first-order rate constant for substrate depletion. This was used to calculate a degradation half-life (T_1/2_), *in vitro* intrinsic clearance (CL_INT_), microsome-predicted blood clearance (CL_blood_) and a microsome-predicted hepatic extraction ratio (E_H_) according to the *in vitro* half-life method of Obach[53].

### Ethics statements

For determination of pharmacokinetic (PK) parameters for NCL812 and NCL195 in mice following systemic exposure, 5-9-week-old male outbred Swiss mice, weighing 21 to 32 g, obtained from Monash Animal Research Platform (MARP), were used. Mice had access to food and water ad libitum throughout the pre- and post-dose sampling period. The Animal Ethics Committee of the Monash Institute of Pharmaceutical Sciences reviewed and approved all animal PK experiments (approval number MIPS.2013.33). The PK studies were conducted in compliance with the Australian Code of Practice for the Care and Use of Animals for Scientific Purposes (7^th^ Edition 2004).

### Determination of PK parameters for NCL812 and NCL195 after systemic exposure

Plasma concentration *vs* time profiles were determined for NCL812 and NCL195 after intravenous (IV) administration at a dose of 5 mg/kg, and for NCL195 after IP administration at a dose of 43 mg/kg. For the IV exposure, each compound was administered via a bolus injection into the tail vein (vehicle 20% (v/v) DMSO in PEG400, 1 ml/kg dose volume, n = 8 mice per compound, resulting in average measured concentration of 5.25 and 4.31 mg/ml for NCL812 and NCL195, respectively). The compounds were administered to mice within 2.5 h of preparation. Following administration, blood samples were collected at 5, 15, 30, 120, 240 and 480 min post- dose (n = 2 mice per time point for each compound). For IP administration of NCL195, the formulation was prepared by dissolving solid NCL195 in DMSO (to 20% (v/v) of the final volume) before addition of PEG400, yielding a clear yellow solution that was dosed to mice within 30 min of preparation. The measured concentration of NCL195 in the final formulation was 21.9 mg/ml, resulting in a mean administered dose of 43 mg/kg, and blood samples were collected up to 24 h post-dose.

For both the IV and IP administration, a maximum of two samples were obtained from each mouse, with samples being taken either via submandibular bleed (approximately 120 μl; conscious sampling) or terminal cardiac puncture (0.6 ml; under inhaled Isoflurane anesthesia). No urine samples were collected as mice were housed in bedded cages during the study. In all experiments, mice were observed continuously for the first hour after dosing, then hourly until up to 6–8 hours after dosing. All animals showed normal behaviour (including grooming, eating, drinking, sleeping, alertness) over the post-dose period. Mice were humanely sacrificed by cervical dislocation while under anaesthesia (using gaseous isoflurane) at the end of experiments. Blood was collected directly into polypropylene Eppendorf tubes containing heparin as anti-coagulant, and stabilization cocktail (containing Complete (a protease inhibitor cocktail with EDTA) and potassium fluoride) to minimize the potential for *ex vivo* degradation of the test compounds in blood/plasma samples. Once collected, blood samples were centrifuged immediately, supernatant plasma was removed, and stored at either -80°C (for the IV exposure) or at -20°C (for the IP exposure) until analysis by liquid chromatography-mass spectrometry (LC-MS). Following protein precipitation with acetonitrile, thawed plasma samples were analyzed by LC-MS (Waters Micromass Quattro Premier coupled to a Waters Acquity UPLC) using positive electrospray ionization operating in MRM mode. PK parameters (apparent half-life, plasma clearance, volume of distribution and area-under-the curve (AUC)) were determined for NCL812 and NCL195 after IV administration using non-compartmental methods (WinNonlin Version 6.3.0.395).

### Haemolysis assay

This was performed using fresh donor human red blood cells (RBCs), essentially as described previously [50]. Fresh RBCs were washed in PBS three times at 500 × *g* for 5 min, and then resuspended 1% (w/v) in PBS. Two microliters of serial 2-fold dilutions of each compound was added into the respective wells, in quadruplicates, in a round bottom 96-well microtitre tray (Sarstedt 82.1582.001), starting at 128 μg/ml for each compound using ampicillin as a control. Thereafter, 198 μL of the 1% RBC solution was added into each well, and the mixture incubated for 1 h at 37°C, with shaking at 100 rpm. Quadruplicate wells containing either 1% Triton X100, or PBS only, served as controls. After incubation, the trays were centrifuged at 1,000 × *g* for 3 min and 100 μl of supernatant from each well transferred into a new 96-well tray. Absorbance was measured at *A*_450 nm_ on a Multiskan Ascent 354 Spectrophotometer (Labsystems) and plotted against each dilution. Haemolytic titer was determined as the reciprocal of the dilution at which 50% of erythrocytes were lysed at *A*_450 nm_. The experiment was performed twice.

### *In vitro* cytotoxicity assays

In order to ensure minimal off target mammalian cell cytotoxicity, we assayed NCL812, NCL195 and NCL219 for *in vitro* cytotoxicity using a panel of adherent mammalian cell lines: Caco-2 (human colorectal adenocarcinoma cell line), HEL 299 (non-cancerous human lung fibroblast cell line), Hep G2 (human hepatocellular carcinoma cell line), MDBK (normal bovine kidney cell line) and MCF7 (human mammary gland adenocarcinoma cell line). Cell lines were maintained in Dulbecco’s Modified Eagle’s Medium (DMEM; Gibco Cat No: 12430) supplemented with 10% (vol/vol) FBS and 1% PenStrep (100 U/ml Penicillin and 100 μg/ml Streptomycin) at 37°C, 5% CO_2_ and passaged ~ every 3 days. Assays were performed in duplicates in flat bottom 96 well tissue culture trays (Sarstedt 83.3924) seeded with either ~2.5 × 10^4^ cells or 5 × 10^4^ cells per well. After 24 h incubation, media was removed, washed once with medium without antibiotics and fresh medium supplemented with 10% (vol/vol) FBS was added. After 2 h, the NCL compounds were diluted in DMSO and added to each well at a concentration of 1% in doubling dilutions starting at the same concentrations used for MIC testing, using wells containing 1% DMSO only as control. Following exposure for 24 h, WST-1 reagent was added to each well at a final concentration of 10%. Absorbance at *A*_450 nm_ on a Multiskan Ascent 354 Spectrophotometer (Labsystems) was recorded after 1 h of incubation. The IC_50_ value for each compound against each cell line was determined via non-linear regression (three parameters) using GraphPad Prism v6 software. The robustness of assays was confirmed with selected cell lines (Hep G2 and MDBK) seeded onto a 96–well Microwell Nunclon Delta Surface plate (Thermo Scientific Nunc; Cat No: 136101) at 5 × 10^4^ cells per well. Viability of each cell line in the presence of 2 or 8 μg/ml of each compound was assessed at 1 h intervals for 20 h at 37°C and 5% CO_2_ on a Cytation 5 Cell Imaging Multi-Mode Reader (BioTek) using the RealTime-Glo MT Cell Viability Assay reagent (Promega). Viability of each cell line in the presence of 2.5, 5, 10 or 20 mM EDTA was also assessed using the same reagent.

## Results

**Fig 1:**
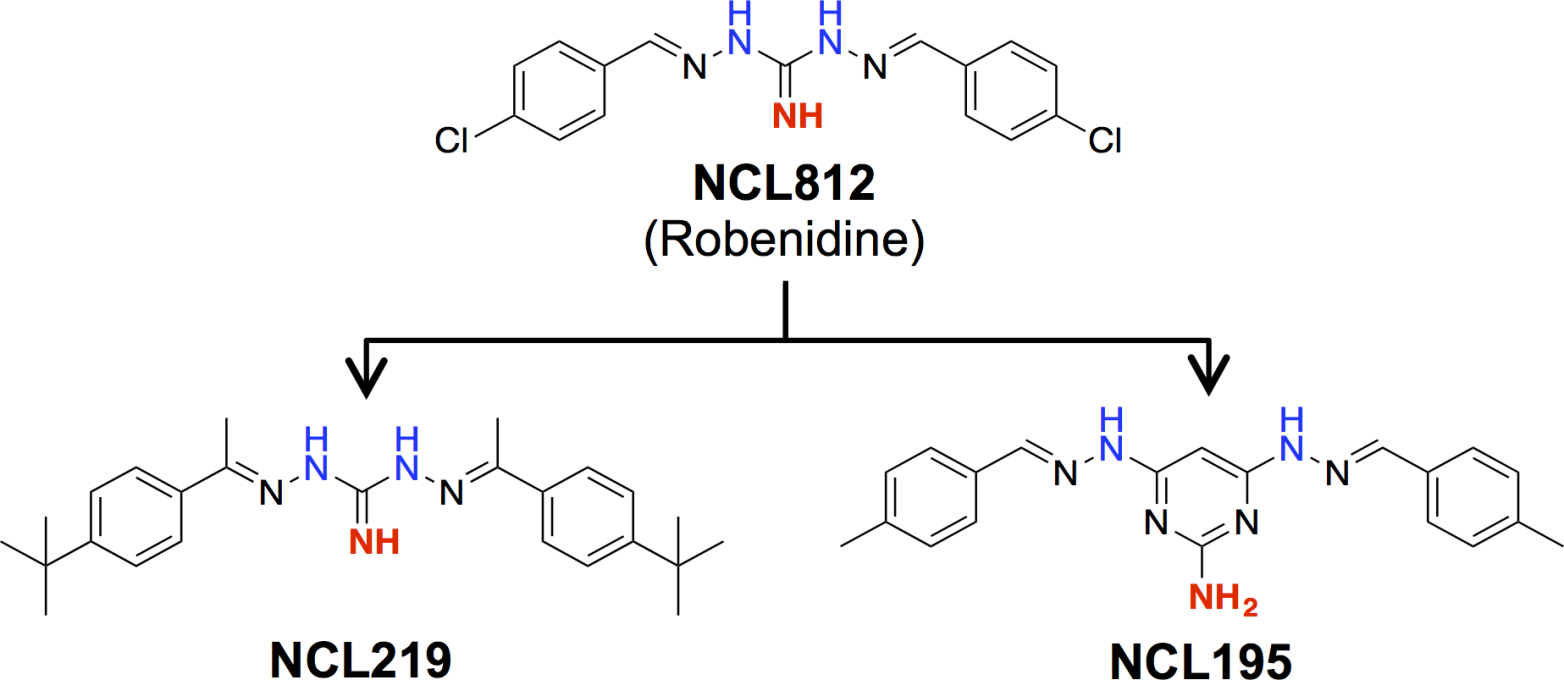
Structure activity relationship between NCL812, NCL195 and NCL219. Installation of a 4-*tert*-butyl and a C-methyl imine moiety provided NCL219 with considerably enhanced hydrolytic stability while retaining the excellent antimicrobial activity of NCL812, while guanidine to 2,4,6-triaminopyrimindine bioisosteric modification yielded NCL195, which allowed potency and drug-like character enhancement.

In this study, building on our previous exploration of the key structure activity relationship of early stage robenidine (NCL812) analogs against *S*. *aureus* and *Enterococcus* spp.,[42] we evaluated the *in vitro* and *in vivo* antibacterial activities of two new analogs in comparison to the parent NCL812 as potential antibacterial agents for both *S*. *pneumoniae* and *S*. *aureus* infection. We developed a series of analogs through condensation of a range of aldehydes and phenones with diaminoguanidine hydrochloride [42]. Subsequent screening for antibacterial activity led to the identification of two contrasting analogs, NCL219 ((*E*)-*N*'-((*E*)-1-(4-(*tert*-butyl)phenyl)ethylidene)-2-(1-(4-(*tert*-butyl)phenyl)ethylidene)hydrazine-1-carboximidhydrazide) and NCL195 (4,6-bis(2-((*E*)-4-methylbenzylidene)hydrazinyl)pyrimidin-2-amine) with the latter resulting from installation of a 2,4,6-triaminopyrimidine as a guanidyl moiety bioisostere (**Fig 1**).

### NCL compounds are bactericidal against *S*. *pneumoniae*, *S*. *aureus* and vancomycin-resistant enterococci

To examine the antimicrobial activities of NCL195, NCL219 and NCL812, we carried out MIC and MBC testing of the three compounds against 21 *S*. *pneumoniae* isolates representing a broad range of serotypes, and 23 clinical *S*. *aureus* isolates of diverse multilocus sequence types. MIC testing of the three compounds was also carried out on 20 vancomycin-resistant enterococcal (VRE) isolates, using *Enterococcus faecalis* ATCC 29212 as a control strain. For *S*. *pneumoniae*, the MIC range for NCL812 and NCL195 were the same (2–8 μg/ml), while the MIC range for NCL219 was 1–8 μg/ml (**Table 1**). In the presence of 10% foetal bovine serum (FBS), the MIC range for the three compounds remained the same (**S1 Table**). For *S*. *aureus*, the MIC range was 2–8 μg/ml for NCL812 (consistent with previous observation with NCL812 [42]; 1–2 μg/ml for NCL195; and 8 - >64 μg/ml for NCL219 (**Table 1**). However, in the presence of 10% FBS, the MIC range increased 4-8-fold to 32 μg/ml for NCL812 (again consistent with previous observation with NCL812 [42]; the MIC range increased 4-fold to 4–8 μg/ml for NCL195, while the MIC range remained essentially the same at 4->64 μg/ml for NCL219 (**S2 Table**). For the VRE isolates, the MIC range for NCL812 and NCL195 were the same (2–4 μg/ml), while the MIC range for NCL219 was 1–2 μg/ml. (**Table 1**). In the presence of 10% FBS, the MIC range increased 8-16-fold to 32 μg/ml for NCL812, the MIC range increased 4-fold to 16–32 μg/ml for NCL195, while the MIC range increased 8-fold to 8–16 μg/ml for NCL219 (**S3 Table**). When the MIC determination for NCL195 against *S*. *pneumoniae* D39 and *S*. *aureus* 49775 was carried out in the presence of human, horse or mouse serum, the MIC remained the same against D39, but a 4-fold increase in MIC was observed against *S*. *aureus* 49775, again as seen previously with NCL812 [42].

**Table 1:** MIC range values for NCL812, NCL195 and NCL219 compared to MIC_90_ values for daptomycin and ampicillin against *S*. *pneumoniae*, *S*. *aureus* and vancomycin resistant enterococci (VRE).

Bacterial strain (No of isolates)	MIC range or MIC_90_ (μg/ml)				
	NCL812	NCL195	NCL219	Daptomycin	Ampicillin
***S*. *pneumoniae* (21)**	2–8	2–8	1–8	0.5	0.5
***S*. *aureus* (23)**	2–8	1–2	8- >64	0.5	>16
**VRE (20)** ^**a**^	2–4	2–4	1–2	2	2

^**a**^ University of South Australia strain collection.

### NCL195 kills *S*. *aureus* without detectable resistance

**Fig 2:**
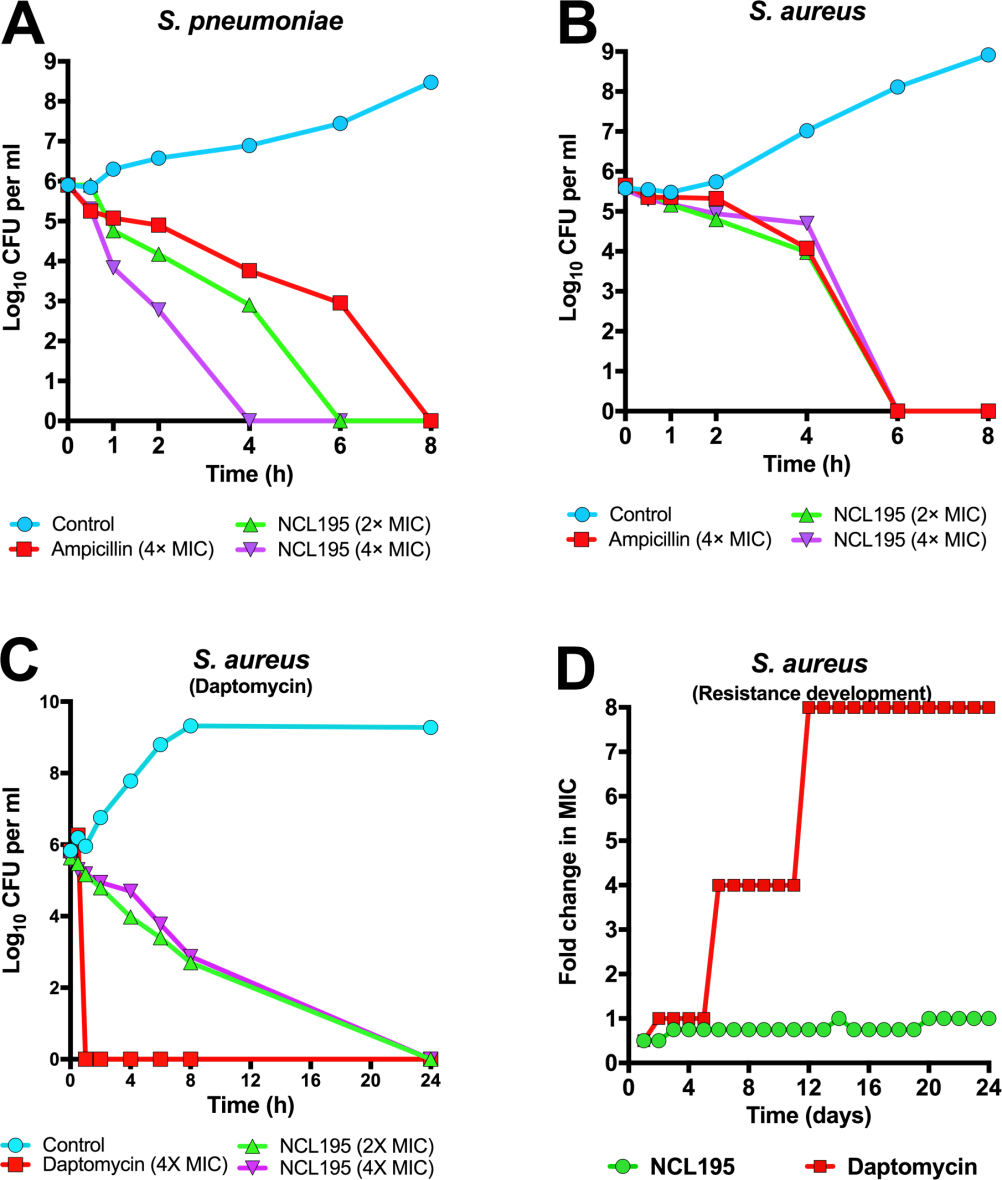
Time kill and point of resistance assays. NCL195 was prepared at 2× and 4× MIC (using 4× MIC of ampicillin (or 4× MIC of daptomycin) and normal saline as controls) in either LB broth supplemented with 3% lysed horse blood and 5% horse serum for *S*. *pneumoniae* D39 (**A**) or LB broth without supplementation for *S*. *aureus* Xen29 (**B and C**). Cultures were incubated statically at 37°C, 5% CO_2_ (for *S*. *pneumoniae* D39) or at 37°C with agitation at 200 rpm (for *S*. *aureus* Xen29). Samples withdrawn at indicated times and plated on HBA overnight at 37°C, 5% CO_2_ (for *S*. *pneumoniae* D39) or at 37°C with aeration (for *S*. *aureus* Xen29) for bacterial enumeration. For (**D**), *S*. *aureus* Xen29 was grown in 2 ml LB broth the presence of 0.5×MIC, 0.75×MIC, 1×MIC, 1.5×MIC, 2×MIC, and 4×MIC of NCL195, using 1×MIC, 4×MIC and 8×MIC of daptomycin as control.

We next performed a kill kinetic assay for NCL812, NCL195 and NCL219 against *S*. *pneumoniae* D39 in a micro-dilution series, with a starting concentration at 64 μg/ml. Our results show that the MBC for the three compounds against *S*. *pneumoniae* D39 was 8, 8 and 4 μg/ml, respectively (equivalent to their respective MIC_90_), confirming that the three compounds are bactericidal against *S*. *pneumoniae*. Similar MIC and MBC results were obtained using *S*. *pneumoniae* D39. For *S*. *aureus* Xen29, the MBC for NCL812, NCL195 and NCL219 was 4, 2 and 16 μg/ml, respectively, confirming their bactericidal activity against another clinically-relevant Gram-positive pathogen. Finally, we interrogated the time-kill profile of NCL195 against either *S*. *pneumoniae* D39 or *S*. *aureus* Xen29, using 2× and 4× MIC of NCL195, with 4× MIC of ampicillin (or 4× MIC of daptomycin) and normal saline serving as controls. We found that it took 6 h to reduce the test population of D39 below the limit of detection at 2× MIC of NCL195; the time-to-clear was reduced to 4 h using 4× MIC of NCL195. Ampicillin at 4× MIC required 8 h to effect clearance of D39 bacteria below the limit of detection (**Fig 2A**). For *S*. *aureus* Xen29, it took 6 h to clear all Xen29 at 2× and 4× MIC of NCL195, and at 4× MIC of ampicillin (**Fig 2B**), while it took only 1 h to clear all Xen29 at 4× MIC of daptomycin (**Fig 2C**). After 24 h, some *S*. *aureus* regrowth was observed for NCL195 and ampicillin at the concentrations tested. We confirmed that the rebound growth was neither due to chemical instability of NCL195 or emergence of a resistant population by adding 10^5^ or 10^3^ c.f.u. of *S*. *aureus* Xen29 in LB broth containing 1×MIC, 2×MIC and 4×MIC of NCL195 over 24–72 h, using daptomycin at either 1×MIC or 4×MIC as control. In this assay, no regrowth was observed in any of the samples upon plating on antibiotic-free plates after 24 h. However, after 72 h incubation in the presence of antibiotic, a few colonies were obtained from broth containing 10^5^ c.f.u. of Xen29 to which 1×MIC, 2×MIC and 4×MIC of NCL195 was added. We subjected the colonies that grew after 72 h to MIC testing and these returned 1× MIC for NCL195.

We then explored the possibility of antimicrobial resistance to NLC195 by performing 24 daily sequential cultures of *S*. *aureus* in the presence of 0.5×MIC, 0.75×MIC, 1×MIC, 1.5×MIC, 2×MIC, and 4×MIC of NCL195, using 1×MIC, 4×MIC and 8×MIC of daptomycin as control. We could not find any resistant mutant above 1× MIC for NCL195 over the 24 passages, although resistance to daptomycin increased up to 8×MIC by day 12 of the serial passage (**Fig 2D**). This was confirmed by MIC testing of the bacteria that grew at the respective MICs.

### NCL195 is bactericidal against Gram-negative bacteria

Our recent studies whereby NCL812 was co-administered with a sub-inhibitory concentration of polymyxin B nonapeptide (a compound known to disrupt the outer membrane of Gram-negative bacteria but with negligible antimicrobial activity) demonstrated Gram-negative activity comparable with clofazimine [42]. We also explored the potential activity of NCL195 against Gram-negative bacteria in the presence or absence of EDTA or polymyxin B, agents also known to disrupt the outer membrane of Gram-negative bacteria [54]. Interestingly, NCL195 in the absence of EDTA was bactericidal against 8 *A*. *calcoaceticus* isolates (MIC range from 4–32 μg/ml) and one *A*. *anitratus* isolate (MIC = 4 μg/ml) (**S4 Table**) and was also bactericidal against *A*. *baumannii*, *E*. *coli*, *K*. *pneumoniae* and *P*. *aeruginosa* (MICs of 0.25–8 μg/ml) in the presence of sub-inhibitory concentrations of EDTA and MICs of 0.25–1 μg/ml in the presence of sub-inhibitory concentrations of polymyxin B (**Table 2**).

**Table 2:** Antimicrobial activity of NCL195 against Gram-negative bacteria alone and in combination with EDTA or polymyxin B.

Bacterial strain	Agent			Combination			
	EDTA (mM)	Polymyxin B (μg/ml)	NCL195 (μg/ml)	EDTA (mM)	NCL 195 (μg/ml)	Polymyxin B (μg/ml)	NCL 195 (μg/ml)
***A*. *baumannii* ATCC 12457**	0.5	0.5	>128	0.25	4	0.125	0.5
***A*. *baumannii* ATCC 19606**	1	0.5	>128	0.5	2	0.125	0.5
***E*. *coli* ATCC 11229**	2.5	0.25	>128	0.625	8	0.125	0.25
***E*. *coli* ATCC 25922**	10	0.25	>128	2.5	4	0.125	0.25
***K*. *pneumoniae* ATCC 4352**	>30	0.25	>128	10	4	0.125	0.5
***K*. *pneumoniae* ATCC 33495**	>30	0.25	>128	10	4	0.125	0.25
***P*. *aeruginosa* ATCC 27853**	10	0.5	>128	5	2	0.125	1
***P*. *aeruginosa* PA01**	2.5	0.5	>128	1.25	0.25	0.125	1

### NCL812 exerts its antibacterial action on the cell membrane of *S*. *pneumoniae* and *S*. *aureus*

**Fig 3:**
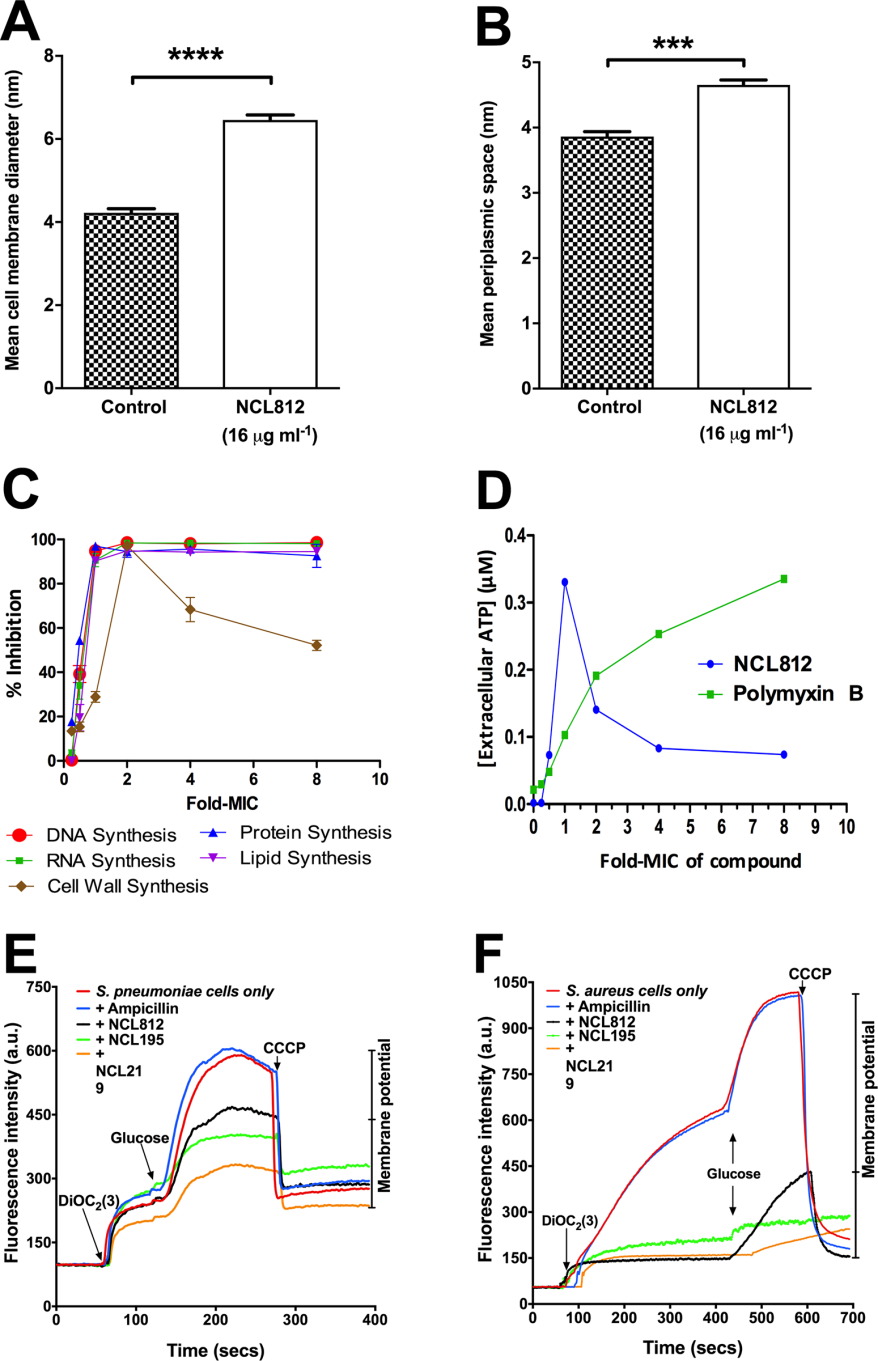
NCL812 compounds exert their antibacterial action on the cell membrane of *S*. *pneumoniae* and *S*. *aureus*. (**A and B**), *S*. *pneumoniae* strain D39 exposed to 16 μg/ml NCL812 for 6 h exhibited significantly thicker cell membranes compared to untreated samples (**A**) (*p* < 0.0001; two-tailed unpaired *t*-test) and displayed significantly wider periplasmic space compared to untreated samples (**B**) (*p* < 0.001; two-tailed unpaired *t*-test. Data presented are an example from 12 different bacterial cells, each with at least 10 measurements per bacteria for both treated and untreated samples. (**C and D**) NCL812 affects macromolecular synthesis (**c**) and ATP release (**D**) in *S*. *aureus*. (**E and F**), NCL Compounds dissipate the membrane potential of *S*. *pneumoniae* and *S*. *aureus*. Membrane potential measurements of *S*. *pneumoniae* D39 (**E**) and *S*. *aureus* ATCC49775 (**F**). Bacterial suspensions were exposed to 16 μg/ml NCL812, NCL195, NCL219, or ampicillin (control) for 5 min after which DiOC_2_(3) was added and the fluorescence monitored until it plateaued. Cells were then re-energized with 0.5% glucose and the establishment of a membrane potential was measured as an increase in fluorescence until it plateaued. The membrane potential was then disrupted by the addition of the proton ionophore (CCCP). Data presented is representative of two experiments. For full description, see Materials and Methods.

To gain further insight into how NCL compounds exert their antibacterial activity on Gram-positive bacteria, we initially examined the effect on the overall cellular architecture and integrity of *S*. *pneumoniae* D39 after 6 h exposure to 16 μg/ml NLC812. Morphometric analysis by transmission electron microscopy (TEM) revealed significant changes to the cell membrane of D39 compared to untreated controls. Treated samples possessed significantly thicker cell membranes (6.43 ± 0.29 nm) compared to untreated samples (4.35 ± 0.24 nm) (*p* < 0.0001) (**Fig 3A**; **Panels A-D in S1 Fig)**. The periplasmic space (intracellular space between the cell membrane and the cell wall) of D39 treated with 16 μg/ml NCL812 was also significantly wider (4.54 ±0.096 nm) compared to untreated bacteria (3.91 ± 0.14 nm) (*p* < 0.001) (**Fig 3B**; **Panels E-H in S1 Fig**). Macromolecular (DNA, RNA, protein, cell wall, and lipid) synthesis inhibition studies on *S*. *aureus* ATCC 29213 exposed to NCL812 also suggested that NCL812 may interact with the cell membrane (**Fig 3C**; **S2 Fig)**, causing leakage of vital metabolites (**Fig 3D**).

To further investigate cell membrane perturbation by the NCL compounds, the ability of *S*. *pneumoniae* and *S*. *aureus* cells to establish a membrane potential (Δψ) when challenged with 16 μg/ml NCL812, NCL195 or NCL219 was determined using the fluorescent membrane potential probe 3,3-diethyloxacarbocyanine iodide (DiOC_2_ (3)). We have previously used this probe to measure the magnitude and stability of Δψ in bacterial cells and proteoliposomes [51]. Bacterial cells were energized by the addition of glucose to establish a proton motive force (negative and basic inside the cell). This led to an increase in fluorescence associated with aggregation of the DiOC_2_ (3). When *S*. *pneumoniae* and *S*. *aureus* were pre-incubated with the NCL compounds, a large reduction in the magnitude of the generated membrane potential compared to that of the untreated cells and cells in the presence of ampicillin was observed (**Fig 3E and 3F**).

### Stability of NCL compounds when incubated with liver microsomes

The metabolic stability of NCL812, NCL195 and NCL219 was evaluated in both human and mouse liver microsomes. NCL812 and NCL195 showed low rates of degradation in both species of liver microsomes (E_H_ values <0.22), with degradation rates for each compound being broadly comparable between species. We did not observe measurable degradation of any of the compounds in control (non-cofactor) incubations in either species suggesting that there was no major cofactor independent metabolism contributing to their overall rates of metabolism. However, NCL219 displayed high rates of degradation in both species of liver microsomes (E_H_ values 0.79 and 0.72, respectively; **Table 3**).

**Table 3:** Physicochemical and metabolism parameters for NCL812, NCL195 and NCL219 in human and mouse liver microsomes.

Properties	Compound					
	NCL812		NCL195		NCL219	
*Physicochemical Parameters*						
Mol Wt^a^	334.20		359.43		405.59	
PSA^a^ (Å^2^)	72.6		100.6		72.6	
FRB^a^	4		6		6	
H-Bond Donor^a^	HBD = 3	HBA = 6	HBD = 4	HBA = 7	HBD = 3	HBA = 5
pKa^a^	5.0; imine (*italics*)	1.2; amine (**bold**)	4.9 / 4.3; amine (*italics*)	2.2; pyrimidine (**bold**)	5.2; imine (**bold**)	2.2 amine (*italics*)
LogD_pH 7. 4_^b^	4.5		4.5		>5.3^d^	
Solubility^c^ (μg/ml)	pH 2.0 = 6.3–12.5	pH 6.5 = <1.6	pH 2.0 = 3.1–6.3	pH 6.5 = <1.6	pH 2.0 = 0.78–1.6	pH 6.5 = 0.78–1.6
*Metabolism parameters*						
Species	Human	Mouse	Human	Mouse	Human	Mouse
T_1/2_ (min)	>247	>247	>247	>247	18	14
CL_INT_ (μl/min/mg protein)	<7	<7	<7	<7	94	121
Predicted CL_blood_ (ml/min/kg)	<5	<16	<5	<16	16	87
Predicted E_H_	<0.22	<0.13	<0.22	<0.13	0.79	0.72

^a^ Calculated using ChemAxon (JChem for Excel).

^b^ Estimated using a gradient HPLC chromatographic method.

^c^ Kinetic solubility estimated via nephelometry.

^d^ the retention times were greater than the most lipophilic standard compound, therefore LogD values are reported as > 5.3.

Mol Wt = molecular weight; PSA = polar surface area; HBD/HBA = hydrogen bond donor / acceptor; pKa is colour coded to the atoms highlighted in each structure. Cl_int_ = *in-vitro* intrinsic clearance; E_H_ = predicted *in-vivo* hepatic extraction ratio.

### NCL compounds exhibit high plasma concentration and low plasma clearance rates

**Fig 4:**
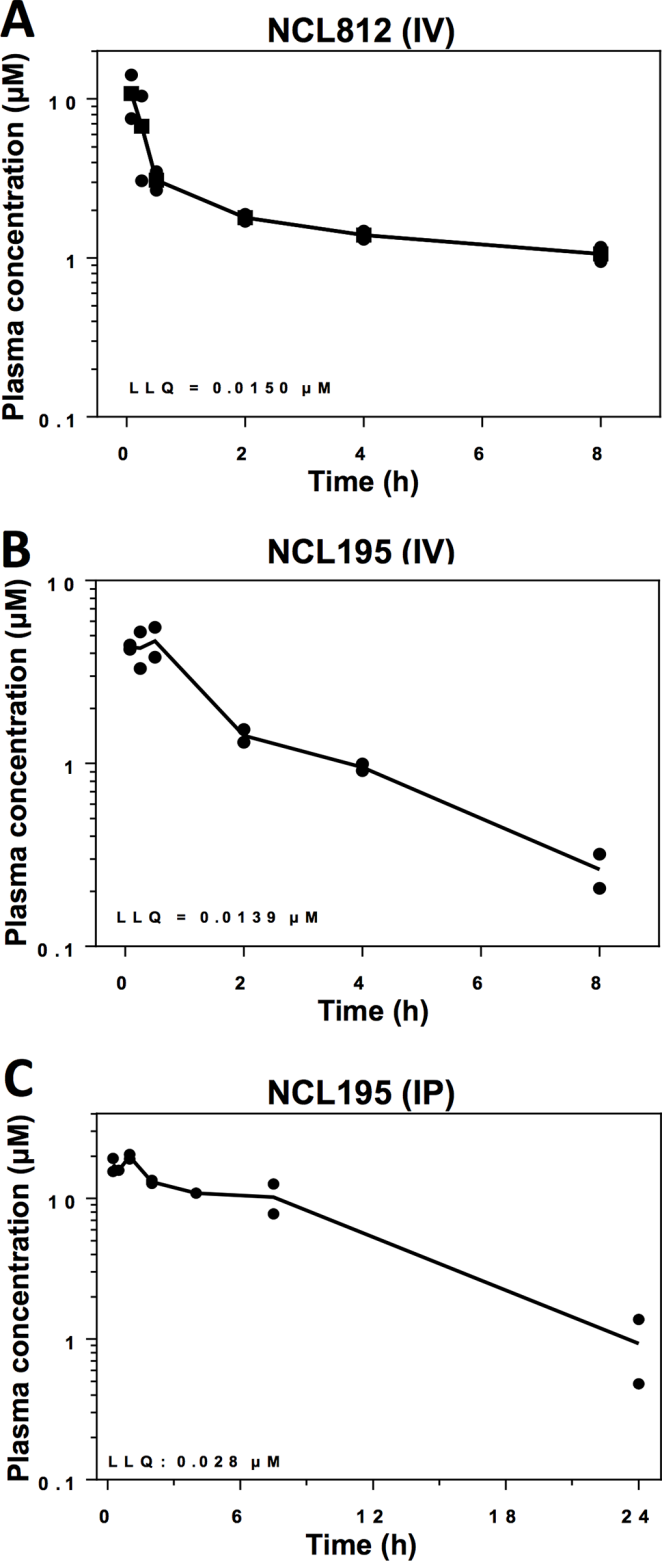
NCL812 and NCL195 exhibit high plasma concentration and low plasma clearance rates. Pharmacokinetic parameters for NCL812 (**A**) and NCL195 (**B**) after IV administration at a dose of 5 mg/kg to male CD1 mice (n = 8 per compound), indicating that both compounds exhibited high plasma concentrations and long apparent terminal elimination half-lives. (**C**), Pharmacokinetic parameters for NCL195 after IP administration at a dose of 43 mg/kg to male CD1 mice (n = 2 per time point), indicating that NCL195 was rapidly absorbed after dosing, and plasma concentrations remained above 3–4 μg/ml for the initial 7.5 h post-dose period.

To examine if NCL812 and NCL195 had appropriate pharmacokinetic (PK) profiles *in vivo* for potential therapeutic use, we evaluated key PK parameters including maximum plasma concentration (Cmax) and apparent terminal elimination half-lives in the plasma of mice after systemic exposure. Our analysis of the PK parameters for NCL812 and NCL195 after IV administration at a dose of 5 mg/kg indicated both compounds exhibited high plasma concentrations (**Fig 4A and 4B**) and long apparent terminal elimination half-lives, with NCL812 showing a higher overall exposure [AUC_0-inf_ 27.9 h*μM (**S5 Table**)]. Furthermore, the estimated plasma clearance values for NCL812 and NCL195 were low (9.5 and 16.3 ml/min/kg, respectively), consistent with the *in vitro* metabolic stability in both human and mouse liver microsomes, and with the microsome-predicted blood clearance values (assuming a blood-to-plasma partitioning ratio close to 1). However, parenteral administration of NCL812 resulted in adverse neurological signs requiring the mice to be anaesthetized for the remainder of the experiment.

We then evaluated the PK parameters of NCL195 after intraperitoneal (IP) administration to Swiss mice at a dose of 43 mg/kg. The plasma concentration-time profile (**Fig 4C**) indicated NCL195 was rapidly absorbed after dosing, and plasma concentrations remained above 3–4 μg/ml for the initial 7.5 h post-dose period, falling to 0.2–0.5 μg/ml between 7.5 and 24 h post-dose. No adverse reactions or compound-related side effects were observed in any of the mice following IP administration of NCL195 at a dose of 43 mg/kg.

### NCL195’s haemolytic activity and cytotoxicity to mammalian cell lines

**Fig 5:**
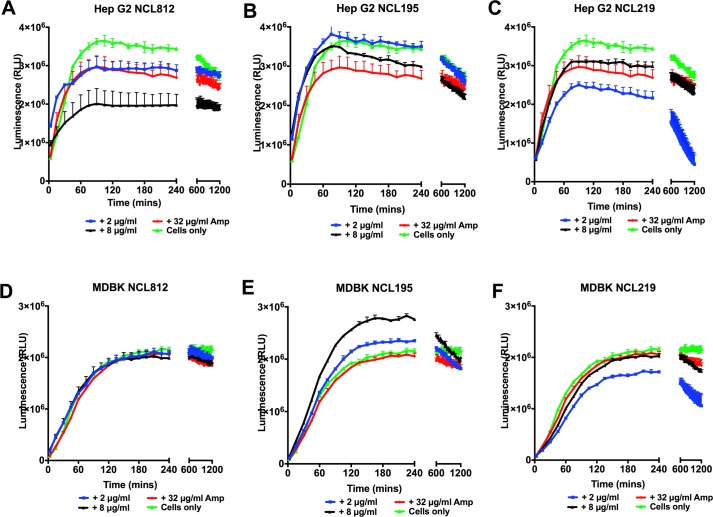
NCL195 demonstrates limited cytotoxicity to mammalian cell lines. Real-time cell viability measurements for Hep G2 (**A, B, C**) and MDBK (**D, E, F**) cells after treatment with 2 or 8 μg/ml NCL812, NCL195 or NCL219. Cell viability was measured every 60 min for 20 h at 37°C and 5% CO_2_ on a Cytation 5 Cell Imaging Multi-Mode Reader (BioTek) using the RealTime-Glo MT Cell Viability Assay reagent (Promega). Data are means (± s.e.m.) relative light units (RLU) for each treatment per time point (in duplicate).

We evaluated the toxicity profile of the three compounds in a panel of normal and carcinoma mammalian cell lines representing different tissue types in order to assess the potential of off-target or dose-limiting toxicity using the WST-1 Cell Proliferation Assay reagent (Roche). The results of the *in vitro* toxicity measurements show IC_50_ values of >4 μg/ml for NCL812, IC_50_ values of >7 μg/ml for NCL219, while the cytotoxicity profile for NCL195 was the most favorable, with IC_50_ values of >10 μg/ml against all the cell lines tested (**S6 Table**). Real-time cell viability measurements using Hep G2 and MDBK cell lines also show no measurable effect on cell viability for NCL195 at either 2 or 8 μg/ml up to 20 h post-treatment (**Fig 5**). However, NCL812 affected viability at 8 μg/ml, while NCL219 had a demonstrable effect on viability at 2 μg/ml.

Haemolytic activity of NCL812, NCL195 and NCL219 against human RBCs showed that the 50% haemolysis titre (HC_50_) for NCL195 was >128 μg/ml (the highest concentration used) and is comparable to that for ampicillin, indicating that NCL195 is well tolerated by RBCs. In contrast, the HC_50_ for NCL812 and NCL219 were 24 and 6 μg/ml, respectively, showing some level of toxicity to human RBCs.

## Discussion

Bacterial pathogens have evolved numerous strategies to adapt to, and thrive in, different environments that they encounter during infection. A major challenge to treatment and control of bacterial pathogens is the emergence and rapid global spread of multidrug-resistant clones that are refractory to last line antimicrobial therapy. To address this problem, we have examined the anticoccidial agent robenidine (NCL812) as a parent scaffold for developing a new antimicrobial class with a potentially novel mechanism of action. Two analogs of NCL812, NCL219 and NCL195, were viewed as promising candidates with the installation of a 4-*tert*-butyl and a C-methyl imine moiety providing NCL219 with considerably enhanced hydrolytic stability while retaining the excellent antimicrobial activity of NCL812. Guanidine to 2,4,6-triaminopyrimindine bioisosteric modification to yield NCL195 allowed potency and drug-like character enhancement. To evaluate their potential as antibacterial agents, we assessed their metabolic stability as well as their pharmacokinetic and safety profiles in a series of *in vitro* and *in vivo* analyses. Furthermore, we assessed *in vitro* efficacy against a range of *S*. *pneumoniae*, *S*. *aureus* and VRE isolates, and examined if activity was extended to Gram-negative bacteria with and without the presence of sub-inhibitory concentrations of EDTA or polymyxin B.

Our preliminary *in vitro* and *in vivo* studies demonstrated NCL812 and NCL195 possess a number of desirable antimicrobial characteristics such as high metabolic stability, prolonged plasma concentration and low plasma clearance rates and fast-acting concentration-dependent bactericidal activity that targets the cell membrane of *S*. *pneumoniae* and *S*. *aureus*. NCL195 showed the most favourable *in vitro* cell cytotoxicity data of the three NCL compounds tested across a range of cell lines, including Caco-2 cells. NCL195’s *in vitro* toxicity data was better across all cell lines to that of the parent compound robenidine (NCL812), an oral anticoccidial drug already approved for veterinary use, and showed no haemolysis of human red blood cells at the highest concentration tested (128 μg/ml). It is possible that *in vitro* cytotoxicity of the NCL compounds is not predictive of *in vivo* toxicity, in agreement with reports by others who note the importance of *in vitro* responses but conclude that the true profile of compound toxicity can only be determined *in vivo* [55–58]. However, NCL812 did elicit adverse clinical signs when administered intravenously to mice at a dose rate of 5mg/kg. Importantly, no compound attributable adverse effects were noted in mice treated with NCL195 which has undergone a major structural modification in the aminoguanidine core compared to NCL812, though further investigation of *in vivo* safety and further medicinal chemistry development is considered desirable and necessary before considering proof of concept *in vivo* efficacy trials in bioluminescent sepsis models.

We previously obtained preliminary data supporting our hypothesis that the site of action of robenidine analogs is present in the Gram-negative bacteria [42], and similar results were obtained for NCL195 in the present study against *N*. *meningitidis* and Gram-negative ESKAPE pathogens such as *Acinetobacter* spp. Importantly, NCL195 in the presence of sub-inhibitory concentrations of EDTA or polymyxin B was active against a variety of additional ESKAPE pathogens. Overall, these are desirable characteristics driving further exploration of robenidine analogs as a novel antimicrobial class to treat acute bacterial infections. The finding that the MICs of NCL195 against strains of *S*. *aureus* and VREs were increased 4-fold in the presence of serum suggests a high protein binding, which may be one of the factors allowing enhanced stability and plasma lifetime without necessarily reducing its effectiveness *in vivo* [59]. As the NCL compounds possess a mechanism of action that targets the cell membrane, they could be more effective than other bactericidal concentration-dependent antimicrobials that have intracellular targets, such as fluoroquinolones and aminoglycosides. Morphometric analysis of the cell membrane and periplasmic space of D39 treated with NCL812 for 6 h showed the cell membrane and periplasmic space was larger in treated samples, compared to control samples. Previous findings with daptomycin [60, 61] and telavancin [62] have described disruption of cell membrane integrity. Our fluorescence membrane potential measurements provide confirmation that the NCL compounds permeabilize the cytoplasmic membrane of *S*. *pneumoniae* and *S*. *aureus*, thereby hindering the establishment and maintenance of essential energy source for cell functioning.

In a comparative resistance selection study of NCL195 and daptomycin, no resistance to NCL195 developed above the MIC for *S*. *aureus* over 24 daily serial passages, whereas resistance to daptomycin developed by day 5, and increased up to 8×MIC by day 12 of the serial passage. Together, these findings demonstrate that NCL195 has potential for further pharmaceutical and medicinal chemistry development to increase solubility, reduce plasma binding and toxicity as well as enhance *in vitro* and *in vivo* potency against leading bacterial pathogens.

## Supporting information

S1 Fig NCL812 exerts its antibacterial action on the cell membrane of *S*. *pneumoniae*. (**A-D**); Comparative visual differences between the cell membranes of treated (**A** and **B**) and untreated (**C** and **D**) *S*. *pneumoniae* D39 samples. The cell membranes of D39 cells exposed to 16 μg/ml NCL812 (**A** and **B**) for 6 h were visually thicker compared to untreated D39 grown for 6 h (**C** and **D**). Length markers represent the cell membrane thickness. (**E-H**); Comparative visual differences in the periplasmic space of treated (**E** and **F**) and untreated (**G** and **H**) *S*. *pneumoniae* D39 samples. The periplasmic space width of D39 cells exposed to 16 μg/ml NCL812 for 6 h (**E** and **F**) was visually larger compared to untreated D39 grown for 6 h (**G** and **H**). Length markers represent the thickness of the periplasmic space. Measurements are representative of 12 bacterial cells for each treatment.
(DOC)

S2 Fig Effect of NCL812 on *Staphylococcus aureus* macromolecular synthesis. NCL812 inhibited DNA (**A**), RNA (**B**), protein (**C**), cell wall (**D**), and lipid (**E**) pathways in exponentially growing culture of *S*. *aureus*, suggesting that NCL812 may interact with the cell membrane. Data are means ± s.e.m. values from triplicate samples for each treatment.
(DOC)

S1 Table MIC values, MIC range, MIC50 and MIC90 (μg/ml) of NCL812, NCL195, and NCL219 for *Streptococcus pneumoniae* isolates in the presence of either 5% or 10% foetal bovine serum with added 3% washed red blood cells. **Each MIC test was performed in duplicate.** ND = Not determined.
(DOCX)

S2 Table MIC values, MIC range, MIC50 and MIC90 (μg/ml) of NCL812, NCL195, and NCL219 for *Staphylococcus aureus* isolates in the absence (-) or presence (+) of 10% foetal bovine serum. **Each MIC test was performed in duplicate.** MLST = Multi-locus Sequence Type; CA = Community Acquired; HA = Healthcare-Associated; MRSA = Methicillin-Resistant *S*. *aureus*; EMRSA = Epidemic Methicillin-Resistant S. aureus; MSSA = Methicillin-Sensitive *S*. *aureus*; PVL = Panton-Valentine Leukocidin status; ND = Not determined.
(DOCX)

S3 Table MIC values, MIC range, MIC50 and MIC90 (μg/ml) of NCL812, NCL195, and NCL219 for porcine vancomycin-resistant enterococci (VRE) in the absence (-) or presence (+) of 10% foetal bovine serum. **Each MIC test was performed in duplicate.** ^a^ Porcine VRE isolates were obtained from The University of South Australia collection. ^b^ *E*. *faecalis* ATCC 29212 was used as a control. ND = Not determined.
(DOCX)

S4 Table MIC values (μg/ml) of NCL195 for *Acinetobacter* spp. (DOCX)

S5 Table Pharmacokinetic parameters for NCL812 and NCL195 in male Swiss outbred mice following IV administration ^a^ Terminal elimination phase was not well defined, value is an approximation only. 8 mice were used per compound.
(DOCX)

S6 Table IC_50_ data for NCL812, NCL195 and NCL219 against a variety of mammalian cell lines. Data presented are mean IC_50_ values from duplicate samples from one experiment. Each experiment was performed twice.
(DOCX)

S1 File Supplementary methods. (DOCX)
