# Supplementary material for: Evaluation of robenidine analog NCL195 as a novel broad-spectrum antibacterial agent
Source: PLoS One. 2017 Sep 5;12(9):e0183457. doi: 10.1371/journal.pone.0183457 (PMC5584945; doi:10.1371/journal.pone.0183457)
Supplement: S1 Fig — (A-D); Comparative visual differences between the cell membranes of treated (A and B) and untreated (C and D) S. pneumoniae D39 samples. The cell membranes of D39 cells exposed to 16 μg/ml NCL812 (A and B) for 6 h were visually thicker compared to untreated D39 grown for 6 h (C and D). Length markers represent the cell membrane thickness. (E-H); Comparative visual differences in the periplasmic space of treated (E and F) and untreated (G and H) S. pneumoniae D39 samples. The periplasmic space width of D39 cells exposed to 16 μg/ml NCL812 for 6 h (E and F) was visually larger compared to untreated D39 grown for 6 h (G and H). Length markers represent the thickness of the periplasmic space. Measurements are representative of 12 bacterial cells for each treatment. (DOC) [file pone.0183457.s001.doc]

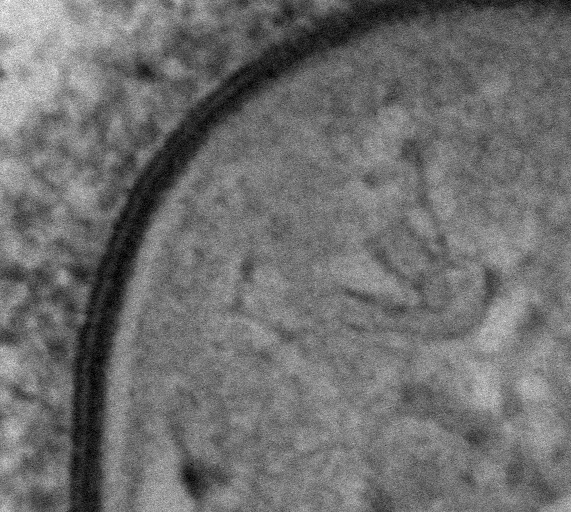

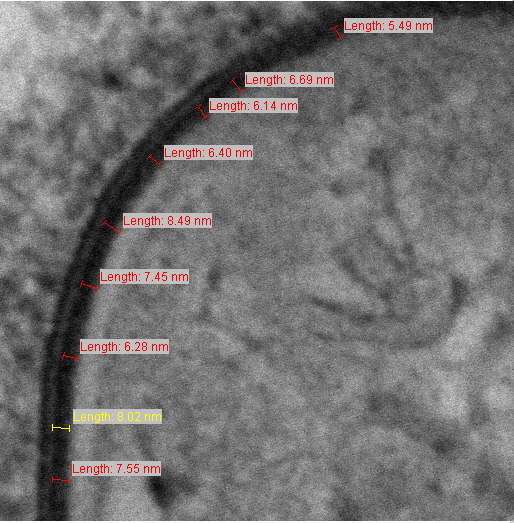

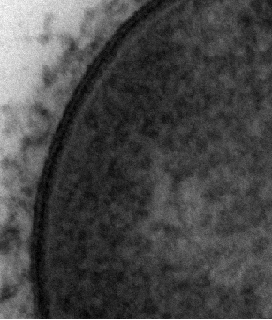

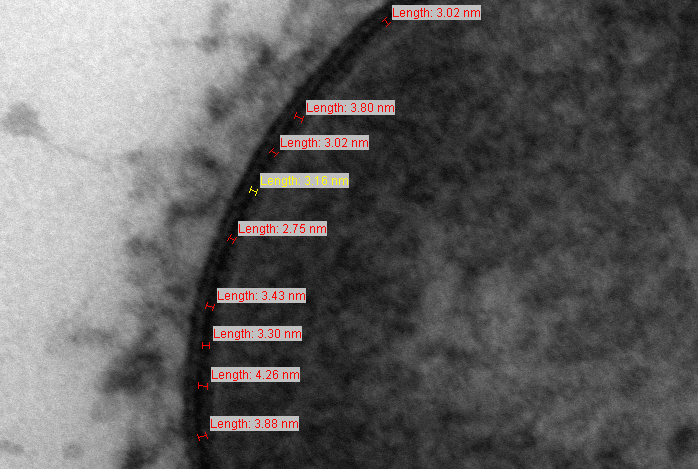

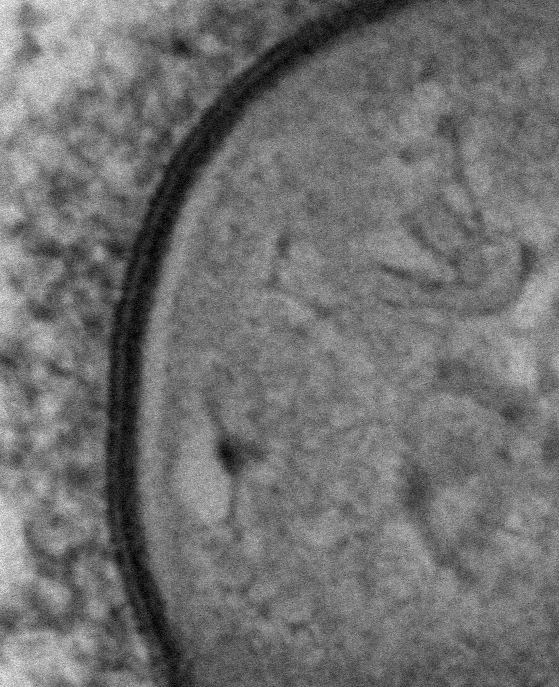

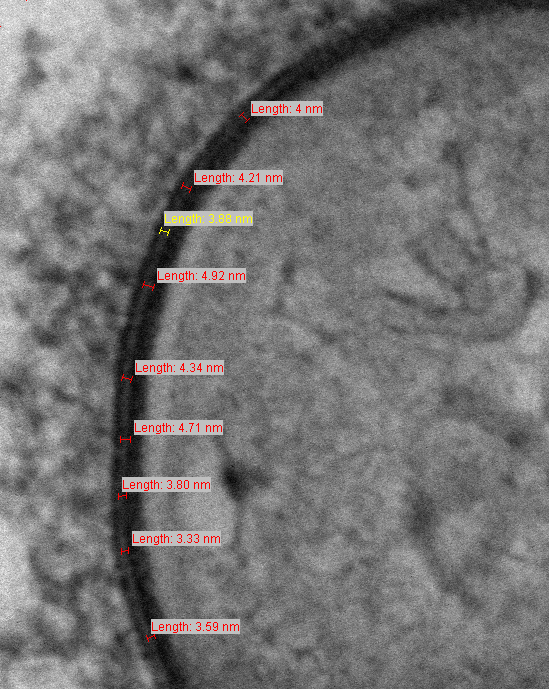

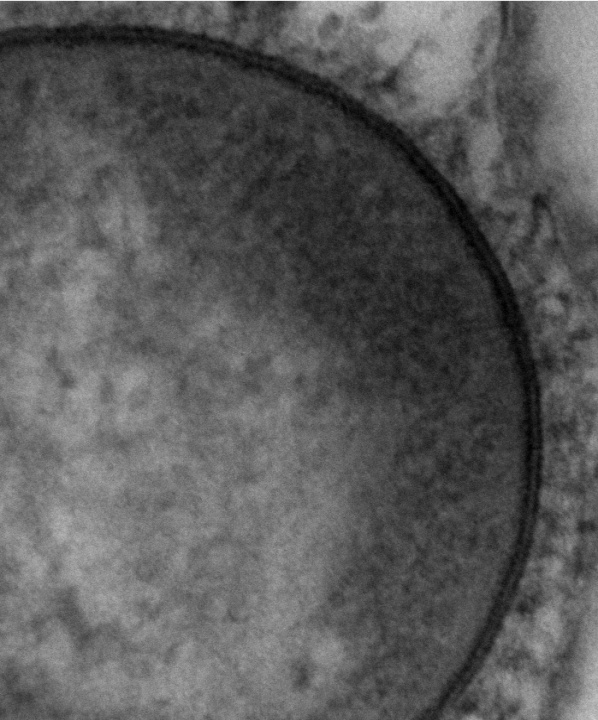

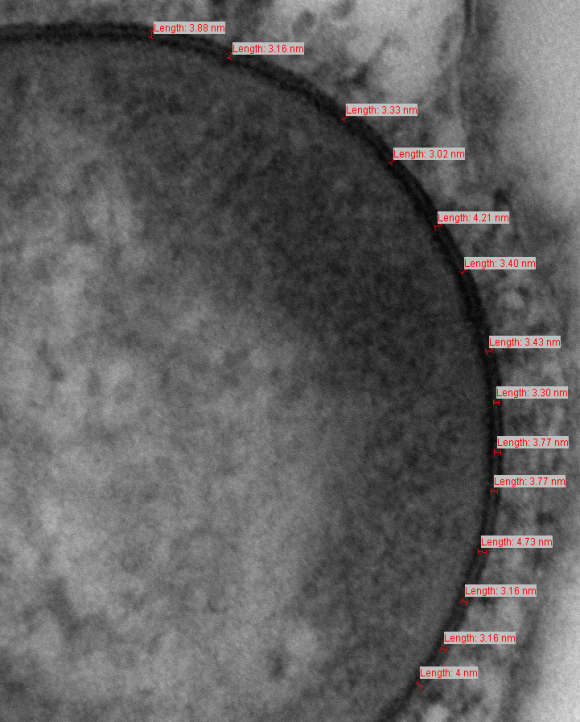


**A**

**B**

**C**

**D**

**E**

**F**

**G**

**H**

**S1 Fig.** **NCL812 exerts its antibacterial action on the cell membrane of *S. pneumoniae*.** (**A-D**); Comparative visual differences between the cell membranes of treated (**a** and **b**) and untreated (**C** and **D**) *S. pneumoniae* D39 samples. The cell membranes of D39 cells exposed to 16 μg/ml NCL812 (**A** and **B**) for 6 h were visually thicker compared to untreated D39 grown for 6 h (**C** and **D**). Length markers represent the cell membrane thickness. (**E-H**); Comparative visual differences in the periplasmic space of treated (**E** and **F**) and untreated (**G** and **H**) *S. pneumoniae* D39 samples. The periplasmic space width of D39 cells exposed to 16 μg/ml NCL812 for 6 h (**E** and **F**) was visually larger compared to untreated D39 grown for 6 h (**G** and **H**). Length markers represent the thickness of the periplasmic space. Measurements are representative of 12 bacterial cells for each treatment.
