# Supplementary material for: Evaluation of robenidine analog NCL195 as a novel broad-spectrum antibacterial agent
Source: PLoS One. 2017 Sep 5;12(9):e0183457. doi: 10.1371/journal.pone.0183457 (PMC5584945; doi:10.1371/journal.pone.0183457)
Supplement: S2 Fig — NCL812 inhibited DNA (A), RNA (B), protein (C), cell wall (D), and lipid (E) pathways in exponentially growing culture of S. aureus, suggesting that NCL812 may interact with the cell membrane. Data are means ± s.e.m. values from triplicate samples for each treatment. (DOC) [file pone.0183457.s002.doc]

**A**

**B**

**C**

**D**

**E**

**S2 Fig.** **Effect of NCL812 on *Staphylococcus aureus* macromolecular synthesis.** NCL812 inhibited DNA (**A**), RNA (**B**), protein (**C**), cell wall (**D**), and lipid (**E**) pathways in exponentially growing culture of *S. aureus*, suggesting that NCL812 may interact with the cell membrane. Data are means ± s.e.m. values from triplicate samples for each treatment.
