# Supplementary material for: Evaluation of robenidine analog NCL195 as a novel broad-spectrum antibacterial agent
Source: PLoS One. 2017 Sep 5;12(9):e0183457. doi: 10.1371/journal.pone.0183457 (PMC5584945; doi:10.1371/journal.pone.0183457)
Supplement: S1 Table — Each MIC test was performed in duplicate. ND = Not determined. (DOCX) [file pone.0183457.s003.docx]

**S1 Table. MIC values, MIC range, MIC50 and MIC90 (μg/ml) of NCL812, NCL195, and NCL219 for *Streptococcus pneumoniae* isolates in the presence of either 5% or 10% foetal bovine serum with added 3% washed red blood cells. Each MIC test was performed in duplicate**.

| ***S. pneumoniae* strain** | **NCL812** | | **NCL195** | | **NCL219** | | **Ampicillin** | | **Daptomycin** | |
| --- | --- | --- | --- | --- | --- | --- | --- | --- | --- | --- |
|  | **5%** | **10%** | **5%** | **10%** | **5%** | **10%** | **5%** | **10%** | **5%** | **10%** |
| ATCC 6303 (serotype 3) | 4 | 4 | 4 | 4 | 1 | 1 | 0.125 | ND | 0.25 | ND |
| P21 (serotype 3) | 4 | 4 | 8 | 8 | 4 | 4 | 0.06 | ND | 0.25 | ND |
| WCH184 (serotype 19F) | 4 | 4 | 8 | 8 | 2 | 2 | 0.125 | ND | 0.25 | ND |
| WCH92 (serotype 4) | 4 | 4 | 4 | 4 | 4 | 4 | 0.5 | ND | 0.125 | ND |
| WCH57 (serotype 8) | 4 | 4 | 4 | 4 | 4 | 4 | 0.5 | ND | 0.25 | ND |
| WCH158 (serotype 19F) | 4 | 4 | 4 | 4 | 8 | 8 | 0.125 | ND | 0.125 | ND |
| WCH77 (serotype 5) | 4 | 2 | 2 | 2 | NA | NA | 0.5 | ND | 0.5 | ND |
| EF3030 (serotype 19F) | 8 | 8 | 8 | 8 | NA | NA | 0.5 | ND | 0.25 | ND |
| P9 (serotype 6A) | 4 | 4 | 8 | 8 | 4 | 4 | 0.06 | ND | 0.25 | ND |
| WCH86 (serotype 4) | 4 | 8 | 4 | 4 | 4 | 4 | 0.06 | ND | 0.125 | ND |
| WCH137 (serotype 6A) | 4 | 4 | 8 | 8 | 4 | 4 | 0.06 | ND | 0.125 | ND |
| L82016 (serotype 6B) | 4 | 4 | 4 | 8 | 2 | 2 | 0.06 | ND | 0.25 | ND |
| TIGR4 (serotype 4) | 4 | 4 | 4 | 4 | 2 | 2 | 0.125 | ND | 0.25 | ND |
| WCH43 (serotype 4) | 4 | 4 | 4 | 4 | 2 | 2 | 0.125 | ND | 0.25 | ND |
| WCH46 (serotype 4) | 4 | 4 | 4 | 4 | 4 | 4 | 0.125 | ND | 0.125 | ND |
| WCH89 (serotype 7) | 4 | 8 | 8 | 8 | 8 | 4 | 0.125 | ND | 0.5 | ND |
| WCH211 (serotype 11) | 4 | 4 | 4 | 4 | 4 | 4 | 0.125 | ND | 0.25 | ND |
| WU2 (serotype 3) | 8 | 8 | 8 | 8 | 4 | 4 | 0.125 | ND | 0.125 | ND |
| WCH16 (serotype 6A) | 4 | 8 | 8 | 8 | 2 | 2 | 1 | ND | 0.25 | ND |
| A66.1 (serotype 3) | 4 | 4 | 8 | 8 | 4 | 4 | 0.125 | ND | 0.25 | ND |
| D39/D39LUX (serotype 2) | 8 | 8 | 8 | 8 | 4 | 4 | 0.125 | ND | 0.25 | 0.25 |
| **MIC range** | 4-8 | 2-8 | 2-8 | 2-8 | 1-8 | 1-8 | 0.06-1 | ND | 0.125-0.5 | ND |
| **MIC_50_** | 4 | 4 | 4 | 8 | 4 | 4 | 0.125 | ND | 0.25 | ND |
| **MIC_90_** | 8 | 8 | 8 | 8 | 4 | 8 | 0.5 | ND | 0.25 | ND |

ND= Not determined.
