# Supplementary material for: Evaluation of robenidine analog NCL195 as a novel broad-spectrum antibacterial agent
Source: PLoS One. 2017 Sep 5;12(9):e0183457. doi: 10.1371/journal.pone.0183457 (PMC5584945; doi:10.1371/journal.pone.0183457)
Supplement: S2 Table — Each MIC test was performed in duplicate. MLST = Multi-locus Sequence Type; CA = Community Acquired; HA = Healthcare-Associated; MRSA = Methicillin-Resistant S. aureus; EMRSA = Epidemic Methicillin-Resistant S. aureus; MSSA = Methicillin-Sensitive S. aureus; PVL = Panton-Valentine Leukocidin status; ND = Not determined. (DOCX) [file pone.0183457.s004.docx]

**S2 Table. MIC values, MIC range, MIC50 and MIC90 (μg/ml) of NCL812, NCL195, and NCL219 for *Staphylococcus aureus* isolates in the absence (-) or presence (+) of 10% foetal bovine serum. Each MIC test was performed in duplicate**.

| ***S. aureus* strain (MLST)** | **NCL812** | | **NCL195** | | **NCL219** | | **Ampicillin** | | **Daptomycin** | |
| --- | --- | --- | --- | --- | --- | --- | --- | --- | --- | --- |
|  | **-** | **+** | **-** | **+** | **-** | **+** | **-** | **+** | **-** | **+** |
| WA1–CA-MRSA (ST1) | 2 | 32 | 1 | 4 | 32 | 8 | >16 | ND | 0.5 | ND |
| WA2–CA-MRSA (ST78) | 4 | 32 | 1 | 4 | >64 | 32 | >16 | ND | 0.5 | ND |
| WA3–CA-MRSA (ST5) | 2 | 32 | 1 | 4 | >64 | 32 | >16 | ND | 0.5 | ND |
| WA84–CA-MRSA (ST45) | 4 | 32 | 1 | 4 | >64 | 8 | >16 | ND | 0.5 | ND |
| QLD PVL^+^ –CA-MRSA (ST93) | 2 | 32 | 2 | 8 | >64 | >64 | >16 | ND | 0.25 | ND |
| AUS2-MRSA (ST239) | 2 | 32 | 2 | 8 | >64 | 8 | >16 | ND | 1.0 | ND |
| AUS3-MRSA (ST239) | 2 | 32 | 1 | 4 | 64 | 8 | >16 | ND | 0.5 | ND |
| Classic–HA-MRSA (ST250) | 2 | 32 | 2 | 8 | 32 | 32 | >16 | ND | 0.5 | ND |
| BENGAL BAY PVL^+^ – CA-MRSA (ST772) | 2 | 32 | 2 | 8 | 16 | 8 | 8 | ND | 0.5 | ND |
| IRISH 1 – HA-EMRSA (ST8) | 2 | 32 | 2 | 8 | >64 | >64 | >16 | ND | 0.5 | ND |
| IRISH 2 – HA-EMRSA (ST8) | 2 | 32 | 1 | 4 | 32 | 8 | >16 | ND | 0.5 | ND |
| UK 15 – HA-EMRSA (ST22) | 4 | 32 | 2 | 8 | 64 | 8 | >16 | ND | 0.5 | ND |
| UK 15 PVL^+^ – HA-EMRSA (ST22) | 4 | 32 | 2 | 8 | 32 | 4 | 16 | ND | 0.5 | ND |
| UK 16 – HA- EMRSA (ST36) | 4 | 32 | 1 | 4 | 32 | 4 | >16 | ND | 0.5 | ND |
| UK 17 – HA-EMRSA (ST247) | 2 | 32 | 2 | 8 | 64 | 16 | >16 | ND | 0.5 | ND |
| TAIWAN PVL^+^ – CA-cMRSA (ST59) | 2 | 32 | 1 | 4 | >64 | 32 | 8 | ND | 0.5 | ND |
| NY JAPAN – HA MRSA (ST5) | 2 | 32 | 1 | 4 | >64 | 4 | >16 | ND | 0.5 | ND |
| WSPP – PVL neg CA MRSA (ST30) | 2 | 32 | 2 | 8 | 32 | 8 | >16 | ND | 0.5 | ND |
| USA 300 - MRSA (ST8) | 2 | 32 | 2 | 8 | >64 | 4 | >16 | ND | 0.5 | ND |
| ST398–CA-MRSA-V (ST398) | 2 | 32 | 2 | 8 | >64 | >64 | >16 | ND | 0.5 | ND |
| ATCC29213 MSSA (ST5) | 4 | 32 | 2 | 8 | 16 | 8 | 2 | ND | 0.5 | ND |
| ATCC 49775 (MSSA) | 2 | 32 | 2 | 8 | 32 | 4 | 0.125 | ND | 0.5 | ND |
| ATCC12600 [Xen29] (MSSA) | 4 | 32 | 1 | 4 | 32 | 4 | 0.125 | ND | 0.5 | 0.5 |
| **MIC range** | 2-8 | 32 | 1-2 | 4-8 | 16->64 | 4->64 | 0.125->16 | ND | 0.25-1  0.5 | ND |
| **MIC_50_** | 2 | 32 | 2 | 8 | 64 | 8 | >16 | ND | 0.5 | ND |
| **MIC_90_** | 4 | 32 | 2 | 8 | >64 | >64 | >16 | ND | 0.5 | ND |

MLST = Multi-locus Sequence Type; CA = Community Acquired; HA = Healthcare-Associated; MRSA = Methicillin-Resistant *S. aureus*; EMRSA = Epidemic Methicillin-Resistant *S. aureus*; MSSA = Methicillin-Sensitive *S. aureus*; PVL = Panton-Valentine Leukocidin status; ND= Not determined.
