# Supplementary material for: Evaluation of robenidine analog NCL195 as a novel broad-spectrum antibacterial agent
Source: PLoS One. 2017 Sep 5;12(9):e0183457. doi: 10.1371/journal.pone.0183457 (PMC5584945; doi:10.1371/journal.pone.0183457)
Supplement: S3 Table — Each MIC test was performed in duplicate. a Porcine VRE isolates were obtained from The University of South Australia collection. b E. faecalis ATCC 29212 was used as a control. ND = Not determined. (DOCX) [file pone.0183457.s005.docx]

**S3 Table. MIC values, MIC range, MIC50 and MIC90 (μg/ml) of NCL812, NCL195, and NCL219 for porcine vancomycin-resistant enterococci (VRE) in the absence (-) or presence (+) of 10% foetal bovine serum. Each MIC test was performed in duplicate**.

| **VRE isolate*^a^*** | **NCL812** | | **NCL195** | | **NCL219** | | **Ampicillin** | | **Daptomycin** | |
| --- | --- | --- | --- | --- | --- | --- | --- | --- | --- | --- |
|  | **-** | **+** | **-** | **+** | **-** | **+** | **-** | **+** | **-** | **+** |
| VRE 1 (49 FR) | 2 | 32 | 4 | 16 | 2 | 16 | 2 | ND | 1 | ND |
| VRE 2 (53 FR) | 2 | 32 | 2 | 16 | 2 | 8 | 1 | ND | 2 | ND |
| VRE 3 (55 FR) | 2 | 32 | 2 | 16 | 2 | 8 | 1 | ND | 2 | ND |
| VRE 4 (57 FR) | 2 | 32 | 2 | 16 | 2 | 8 | 1 | ND | 2 | ND |
| VRE5 (58 FR) | 4 | 32 | 4 | 32 | 2 | 8 | 1 | ND | 2 | ND |
| VRE 6 (59 FR) | 2 | 32 | 4 | 16 | 2 | 8 | 2 | ND | 2 | ND |
| VRE 7 (270 G) | 4 | 32 | 4 | 32 | 2 | 16 | 0.25 | ND | 1 | ND |
| VRE 8 (277 G) | 2 | 32 | 4 | 32 | 1 | 8 | 2 | ND | 0.5 | ND |
| VRE 9 (61 FR) | 2 | 32 | 4 | 16 | 1 | 8 | 1 | ND | 1 | ND |
| VRE 10 (62 FR) | 2 | 32 | 4 | 16 | 1 | 16 | 2 | ND | 2 | ND |
| VRE 11 (284 G) | 2 | 32 | 4 | 16 | 2 | 8 | 2 | ND | 0.5 | ND |
| VRE 12 (290 G) | 2 | 32 | 2 | 16 | 2 | 8 | 1 | ND | 1 | ND |
| VRE 13 (251 G) | 2 | 32 | 2 | 16 | 2 | 8 | 1 | ND | 0.5 | ND |
| VRE 14 (252 G) | 2 | 32 | 2 | 32 | 2 | 8 | 0.5 | ND | 1 | ND |
| VRE 15 (63 FR) | 2 | 32 | 2 | 16 | 2 | 8 | 1 | ND | 1 | ND |
| VRE 16 (60 FR) | 4 | 32 | 2 | 16 | 2 | 8 | 1 | ND | 2 | ND |
| VRE 17 (45 FR) | 2 | 32 | 2 | 32 | 2 | 8 | 2 | ND | 2 | ND |
| VRE 18 (69 W) | 2 | 32 | 2 | 16 | 2 | 8 | 1 | ND | 1 | ND |
| VRE 19 (70 W) | 2 | 32 | 2 | 16 | 2 | 8 | 1 | ND | 0.5 | ND |
| VRE 20 (78 W) | 2 | 32 | 4 | 32 | 2 | 8 | 1 | ND | 2 | ND |
| *Enterococcus faecalis* ATCC 29212***^b^*** | 4 | 32 | 4 | 32 | 2 | 8 | 2 | ND | 2 | 2 |
| **MIC range** | 2-4 | 32 | 2-4 | 16-32 | 1-2 | 8-16 | 0.25-2 | ND | 0.5-2 | ND |
| **MIC_50_** | 2 | 32 | 2 | 16 | 2 | 8 | 1 | ND | 1 | ND |
| **MIC_90_** | 2 | 32 | 4 | 32 | 2 | 16 | 2 | ND | 2 | ND |

***^a^*** Porcine VRE isolates were obtained from The University of South Australia collection.

***^b^*** *E. faecalis* ATCC 29212 was used as a control.

ND= Not determined.
