# Supplementary material for: Evaluation of robenidine analog NCL195 as a novel broad-spectrum antibacterial agent
Source: PLoS One. 2017 Sep 5;12(9):e0183457. doi: 10.1371/journal.pone.0183457 (PMC5584945; doi:10.1371/journal.pone.0183457)
Supplement: S4 Table — (DOCX) [file pone.0183457.s006.docx]

**S4 Table.** MIC values (μg/ml) of NCL195 for *Acinetobacter* spp.

| **Bacterial isolates** | **MIC (μg/ml) of NCL195** |
| --- | --- |
| *A. baumannii* ATCC19606 | >128 |
| *A. baumannii* ATCC 12457 | >128 |
| *A. baumannii* | >128 |
| *A. baumannii* | >128 |
| *A. baumannii* | >128 |
| *A. baumannii* | >128 |
| *A. baumannii* | >128 |
| *A. baumannii* | >128 |
| *A. baumannii* | >128 |
| *A. baumannii* | >128 |
| *A. baumannii* | >128 |
| *A. baumannii* | >128 |
| *A. baumannii* | >128 |
| *A. baumannii* | >128 |
| *A. baumannii* | >128 |
| *A. baumannii* | >128 |
| *A. baumannii* | >128 |
| *A. baumannii* | >128 |
| *A. calcoaceticus* | 4 |
| *A. calcoaceticus* | 16 |
| *A. calcoaceticus* | 32 |
| *A. calcoaceticus* | 4 |
| *A. calcoaceticus* | 4 |
| *A. calcoaceticus* | 16 |
| *A. calcoaceticus* | 4 |
| *A. calcoaceticus* | 4 |
| *A. anitratus* | 4 |
