# Supplementary material for: Evaluation of robenidine analog NCL195 as a novel broad-spectrum antibacterial agent
Source: PLoS One. 2017 Sep 5;12(9):e0183457. doi: 10.1371/journal.pone.0183457 (PMC5584945; doi:10.1371/journal.pone.0183457)
Supplement: S5 Table — a Terminal elimination phase was not well defined, value is an approximation only. 8 mice were used per compound. (DOCX) [file pone.0183457.s007.docx]

**S5 Table.** Pharmacokinetic parameters for NCL812 and NCL195 in male Swiss outbred mice following IV administration.

| **Parameter** | **NCL812** | **NCL195** |
| --- | --- | --- |
| Measured dose (mg/kg) | 5.3 | 4.4 |
| Apparent t_½_ (h) | 8.2 ^a^ | 2.4 |
| Plasma CL (mL/min/kg) | 9.5 ^a^ | 16.3 |
| Plasma V_SS_ (L/kg) | 5.9 ^a^ | 2.6 |
| AUC_0-inf_ (h*μM) | 27.9 ^a^ | 12.5 |

^a^ Terminal elimination phase was not well defined, value is an approximation only. 8 mice were used per compound.
