# Supplementary material for: Evaluation of robenidine analog NCL195 as a novel broad-spectrum antibacterial agent
Source: PLoS One. 2017 Sep 5;12(9):e0183457. doi: 10.1371/journal.pone.0183457 (PMC5584945; doi:10.1371/journal.pone.0183457)
Supplement: S6 Table — Data presented are mean IC50 values from duplicate samples from one experiment. Each experiment was performed twice. (DOCX) [file pone.0183457.s008.docx]

**S6 Table.** IC_50_ data for NCL812, NCL195 and NCL219 against a variety of mammalian cell lines.

| **Cell line** | **IC_50_ values ± SEM** **(μg/ml) for:** | | |
| --- | --- | --- | --- |
|  | **NCL812** | **NCL195** | **NCL219** |
| Caco-2 | 8.2 ± 0.1 | 12.8 ± 0.1 | 7.5 ± 0.1 |
| HEL 299 | 4.6 ± 0.1 | 10.9 ± 0.2 | 21.2 ± 0.2 |
| Hep G2 | 9.4 ± 0.1 | 12.0 ± 0.1 | 7.1 ± 0.1 |
| MCF7 | 12.0 ± 0.2 | 40.1 ± 0.2 | >128 |
| MDBK | 5.9 ± 0.1 | 17.3 ± 0.2 | 16.0 ± 0.2 |

Data presented are mean **± SEM** IC_50_ values from duplicate samples from one experiment. Each experiment was performed twice.
