# Supplementary material for: Evaluation of robenidine analog NCL195 as a novel broad-spectrum antibacterial agent
Source: PLoS One. 2017 Sep 5;12(9):e0183457. doi: 10.1371/journal.pone.0183457 (PMC5584945; doi:10.1371/journal.pone.0183457)
Supplement: S1 File — (DOCX) [file pone.0183457.s009.docx]

**S1 File. Supplementary Methods.**

**Effect of NCL812 on *S. pneumoniae* D39 cell membrane ultra-structure.** Morphological appearance and morphometric analysis of the cell membrane of *S. pneumoniae* D39 after exposure to either 1 μg/ml, 4 μg/ml or 16 μg/ml of NCL812 was determined using TEM. For this assay, 10 ml cultures of D39 were prepared and incubated at 37°C, 5% CO_2_ with gentle manual tilting of the cultures every 10 min, due to the insolubility of NCL812. Cultures were then exposed to either 1 μg/ml, 4 μg/ml or 16 μg/ml of NCL812 and harvested at 6 or 12 h by centrifugation at 100 × *g* for 20 min and washed twice in 50 ml PBS. Critical time points for TEM work were determined by analyzing trends in the growth curves produced from the kill kinetics studies. Samples were resuspended in PBS containing 20% glycerol and stored at -80°C until required. Before fixation, 20% glycerol was removed by centrifugation and cells were washed on ice three times in 50 ml PBS.

Samples were fixed using modified protocols defined by a previous study examining cell wall ultrastructure of *S. pneumoniae* [[1](#_ENREF_1)]. Briefly, a lysine-acetate-based formaldehyde-glutaraldehyde ruthenium red-osmium fixation procedure involved fixing the bacterial pellets with a cacodylate buffer solution containing 2% formaldehyde, 2.5% glutaraldehyde, 0.075% ruthenium red and 0.075 M of lysine acetate, was used. Samples were washed and dehydrated using a graded series of ethanol (70, 90, 95 and 100%) for 10-20 min, two times for each step. Samples were infiltrated using 50:50 LR White resin in 100% ethanol for 1 h, and subsequently washed with 100% LR White resin for 1 h and left O/N in a third change of 100% LR white to ensure adequate infiltration of resin. The samples were then embedded in fresh LR White resin and incubated at 50°C for 48 h. Sections were cut to 1 μm using a glass knife, stained with Toluidene Blue and viewed under a light microscrope at 400× to identify the presence of stained pneumococci. Ultra-thin sections were then stained with uranyl acetate and lead citrate alternatively at 5 min intervals, followed by three washes with distilled water in-between each exposure. Stained sections were then placed on grids and viewed between 25000× and 130000× on a Philips CM100 Transmission Electron Microscope. Images were obtained at 130000× magnification and analyzed using analySIS (Olympus Soft Imaging Systems).

**Effect of NCL812 on *S. aureus* ATCC29213 macromolecular synthesis**. Macromolecular (DNA, RNA, protein, cell wall, and lipid) synthesis inhibition studies were performed under external contract to Micromyx LLC, Kalamazoo, MI, USA 49008, as follows. Prior to these assays, *S. aureus* ATCC 29213 were grown to early exponential growth phase (*A*_600 nm_ = 0.2 to 0.3) in Mueller Hinton broth II (MHBII) at 35^o^C with shaking at 200 rpm.

For DNA, RNA and protein synthesis, 100 μl of the culture was added to triplicate wells containing various concentrations of NCL812 that were equivalent to 0, 0.25, 0.5, 1, 2, 4 or 8-fold the MIC value (4 μg/ml) for *S. aureus* ATCC 29213 or control antibiotics (5 μl) at 20X the final concentration in 100% DMSO. A 5% DMSO treated culture served as the “no drug” control for all experiments. Cells were added in MHBII at 105% to account for the volume of drug added to each reaction or in M9 minimal medium for protein synthesis reactions. Following a 15 min incubation at room temperature, either [^3^H] thymidine (DNA synthesis), [^3^H] uridine (RNA synthesis) or [^3^H] leucine (protein synthesis) was added at 0.5-1.0 μCi per reaction, depending on the experiment. Reactions were allowed to proceed at room temperature for 15-30 min and then stopped by adding 12 μl of cold 5% trichloroacetic acid (TCA) or 5% TCA/2% casamino acids (protein synthesis only). Reactions were incubated on ice for 30 min and the TCA precipitated material was collected on a 25 mm GF/A filter. After washing three times with 5 ml of cold 5% TCA, the filters were rinsed two times with 5 ml 100% ethanol, allowed to dry, and then counted using a Beckman LS3801 liquid scintillation counter.

To test the effect of NCL812 on cell wall synthesis, bacteria were transferred to M9 minimal medium and added to 1.5 ml eppendorf tubes (100 μl/tube) containing various concentrations of test compound or control antibiotics (5 μl) at 20X the final concentration in 100% DMSO as described above. Following a 5 min incubation at 37^o^C, [^14^C]N-acetylglucosamine (0.4 μCi/reaction) was added to each tube and incubated for 45 min in a 37^o^C heating block. Reactions were stopped through the addition of 100 μl of 8% SDS to each tube. Reactions were then heated at 95^o^C for 30 min in a heating block, cooled, briefly centrifuged, and spotted onto pre-wet HA filters (0.45 μM). After washing three times with 5 ml of 0.1% SDS, the filters were rinsed two times with 5 ml of deionized water, allowed to dry, and then counted using a Beckman LS3801 liquid scintillation counter.

To assay for the effect of NCL812 on lipid synthesis, bacteria were added to 1.5 ml eppendorf tubes (in triplicate) containing various concentrations of test compound or control antibiotics as described above. Following a 5 min incubation at room temperature, [^3^H]glycerol was added at 0.5 μCi per reaction. Reactions were allowed to proceed at room temperature for 15 min and then stopped through the addition of 375 μl chloroform/methanol (1:2) followed by vortexing for 20 seconds after each addition. Chloroform (125 μl) was then added to each reaction, vortexed, followed by the addition of 125 μl dH_2_O and vortexing. Reactions were centrifuged at 13,000 rpm for 10 min, and then 150 μl of the organic phase was transferred to a scintillation vial and allowed to dry in a fume hood for at least 1 h. Samples were then assayed in a liquid scintillation counter.

In order to measure the leakage of ATP from the bacteria, the CellTiter-Glo Luminescent Cell Viability Assay (Promega) was used. The bacteria were grown to exponential phase as described above and then treated with seven different concentrations of either NCL812 or polymyxin B (positive control) utilizing the MIC for each compound as a guide (0, 0.25, 0.5, 1, 2, 3, 4, or 8-fold the MIC). The negative control received 2% DMSO, which represented the final DMSO concentration in each assay. After a 30 min exposure to drug, cells were centrifuged and the supernatant was analyzed for the presence of ATP. Results were expressed as ATP concentration released to the medium (μM).

1. Hammerschmidt S, Wolff S, Hocke A, Rosseau S, Muller E, Rohde M. Illustration of pneumococcal polysaccharide capsule during adherence and invasion of epithelial cells. Infect Immun. 2005;73(8):4653-67. Epub 2005/07/26. doi: 73/8/4653 [pii]

10.1128/IAI.73.8.4653-4667.2005. PubMed PMID: 16040978; PubMed Central PMCID: PMC1201225.
